# Historical biogeography, systematics, and integrative taxonomy of the non-Ethiopian speckled pelage brush-furred rats (*Lophuromys flavopunctatus* group)

**DOI:** 10.1186/s12862-021-01813-w

**Published:** 2021-05-19

**Authors:** Kenneth Otieno Onditi, Terrence C. Demos, Julian Kerbis Peterhans, Zhong-Zheng Chen, Josef Bryja, Leonid A. Lavrenchenko, Simon Musila, Erik Verheyen, Frederik Van de Perre, Benjamin Dudu Akaibe, Noé U. de la Sancha, Xue-Long Jiang

**Affiliations:** 1 State Key Laboratory of Genetic Resources and Evolution, Kunming Institute of Zoology, Chinese Academy of Sciences, Kunming, China; 2 Kunming College of Life Science, University of Chinese Academy of Sciences, Kunming, China; 3 Mammal Section, Zoology Department, National Museums of Kenya, Nairobi, Kenya; 4 Science & Education, Field Museum of Natural History, Chicago, USA; 5 College of Arts and Sciences, Roosevelt University, Chicago, USA; 6 Collaborative Innovation Centre of Recovery and Reconstruction of Degraded Ecosystems in Wanjiang Basin Co-founded by Anhui Province and Ministry of Education, School of Ecology and Environment, Anhui Normal University, Anhui, China; 7 Institute of Vertebrate Biology, Czech Academy of Sciences, Brno, Czech Republic; 8 Department of Botany and Zoology, Faculty of Science, Masaryk University, Brno, Czech Republic; 9 A.N. Severtsov Institute of Ecology and Evolution, Russian Academy of Science, Moscow, Russia; 10 Sino-Africa Joint Research Centre, Chinese Academy of Sciences, Nairobi, Kenya; 11 Operational Direction Taxonomy and Phylogeny, Royal Belgian Institute for Natural Sciences, Brussels, Belgium; 12 Evolutionary Ecology Group, Department of Biology, University of Antwerp, Antwerp, Belgium; 13 Department of Ecology and Animal Resource Management, Faculty of Science, Biodiversity Monitoring Centre, University of Kisangani, Kisangani, Democratic Republic of the Congo; 14 Department of Biological Sciences, Chicago State University, Chicago, USA

**Keywords:** East Africa, *Kivumys*, *Lophuromys flavopunctatus* group, *Lophuromys*, Biogeography, Integrative systematics

## Abstract

**Background:**

The speckled-pelage brush-furred rats (*Lophuromys flavopunctatus* group) have been difficult to define given conflicting genetic, morphological, and distributional records that combine to obscure meaningful accounts of its taxonomic diversity and evolution. In this study, we inferred the systematics, phylogeography, and evolutionary history of the *L. flavopunctatus* group using maximum likelihood and Bayesian phylogenetic inference, divergence times, historical biogeographic reconstruction, and morphometric discriminant tests. We compiled comprehensive datasets of three loci (two mitochondrial [mtDNA] and one nuclear) and two morphometric datasets (linear and geometric) from across the known range of the genus *Lophuromys*.

**Results:**

The mtDNA phylogeny supported the division of the genus *Lophuromys* into three primary groups with nearly equidistant pairwise differentiation: one group corresponding to the subgenus *Kivumys* (*Kivumys* group) and two groups corresponding to the subgenus *Lophuromys* (*L. sikapusi* group and *L. flavopunctatus* group). The *L. flavopunctatus* group comprised the speckled-pelage brush-furred *Lophuromys* endemic to Ethiopia (Ethiopian *L. flavopunctatus* members [ETHFLAVO]) and the non-Ethiopian ones (non-Ethiopian *L. flavopunctatus* members [NONETHFLAVO]) in deeply nested relationships. There were distinctly geographically structured mtDNA clades among the NONETHFLAVO, which were incongruous with the nuclear tree where several clades were unresolved. The morphometric datasets did not systematically assign samples to meaningful taxonomic units or agree with the mtDNA clades. The divergence dating and ancestral range reconstructions showed the NONETHFLAVO colonized the current ranges over two independent dispersal events out of Ethiopia in the early Pleistocene.

**Conclusion:**

The phylogenetic associations and divergence times of the *L. flavopunctatus* group support the hypothesis that paleoclimatic impacts and ecosystem refugia during the Pleistocene impacted the evolutionary radiation of these rodents. The overlap in craniodental variation between distinct mtDNA clades among the NONETHFLAVO suggests unraveling underlying ecomorphological drivers is key to reconciling taxonomically informative morphological characters. The genus *Lophuromys* requires a taxonomic reassessment based on extensive genomic evidence to elucidate the patterns and impacts of genetic isolation at clade contact zones.

**Supplementary Information:**

The online version contains supplementary material available at 10.1186/s12862-021-01813-w.

## Background

Correctly resolving species taxonomic and biogeographic accounts enable accurate species delimitation, providing an objective framework for useful biodiversity quantification and management [1]. While faunas with high abundance and richness in high biodiversity areas are key to untangling how ecological interactions drive evolutionary processes in these areas, their taxonomy is commonly confounded by conflicting morphological descriptions and scarce molecular accounts. New developments in integrative morphologic, phylogeographic, genetic, and ecological analysis have increasingly complemented traditional reliance on morphological evidence to resolve taxonomic limits [2]. This ‘integrative systematics’ approach is most effective when delimiting cryptic species [3].

The genus *Lophuromys* contains between 15 and 34 recognized species, with the variable number attributed to debatable morphological differences between species [4–7]. The genus was placed in the Murinae subfamily until recently when Steppan et al*.* [8] and Steppan et al*.* [9] noted genetic affinity between *Lophuromys, Uranomys, Deomys,* and *Acomys* that warranted their classification as a unique subfamily—Deomyinae. In deep phylogenetic relationships, morphology divides the genus into two subgenera; *Lophuromys*, with shorter tails and hindfeet, and *Kivumys*, with longer tails and hindfeet and unique gastrointestinal morphology [10, 11]. In the subgenus *Lophuromys*, three species groups have been defined based on pelage coloration and craniodental characterization; the *L. sikapusi* group with unspeckled dorsal pelage, the *L. flavopunctatus* group and *L. aquilus* group, both with speckled dorsal pelage coloration [6, 12]. Between the two speckled-pelage groups—*L. flavopunctatus* group and *L. aquilus* group—species are classified based on morphological affinities. However, it is not clear how morphology (external body features, pelage color, craniodental characters) explicitly delimits species in the literature [6, 12]. Some Ethiopian endemics, such as *L. brunneus* and *L. chrysopus*, are included in the mainly non-Ethiopian *L. aquilus* group [12–14] while the inclusion of the unspeckled-pelage *L. dieterleni* and *L. eisentrauti* in the *L. aquilus* group and *L. pseudosikapusi* in the *L. flavopunctatus* group [6] confounds further how pelage coloration relates to phylogenetic relationships. There is a need to clearly define whether and how pelage coloration and morphological affinities relate to phylogenetic relationships in the genus *Lophuromys*. Hereafter, we use ‘Ethiopian *L. flavopunctatus* members [ETHFLAVO]’ to refer to the *Lophuromys* taxa endemic to the Ethiopian Highlands, the ‘non-Ethiopian *L. flavopunctatus* members [NONETHFLAVO]’ to refer to the remaining *Lophuromys* taxa not belonging to the *L. sikapusi* group or the *Kivumys* group, while the ‘*L. flavopunctatus* group’ is used to combine ETHFLAVO and NONETHFLAVO.

In contrast to the relatively resolved taxonomy of the ETHFLAVO [15–17], the NONETHFLAVO generally lacks broader phylogenetic and biogeographical understanding. A chronological review of the genus *Lophuromys* reveals persistent taxonomic controversy, especially concerning the morphological traits used to diagnose species, synonyms, and species groups [6]. Such controversy is most notable in the descriptions of several new species in checklists compiled before the twenty-first century, which relied exclusively on external morphology and craniodental characters for taxonomic designations [18–21]. Checklists compiled in the twenty-first century, employing more integrative techniques, also vary in the individual number of species recognized, and generally agree on an increasing number of recognized species, ranging from 21 species [6] to 15 species [22], and most recently 34 species [5, 7]. The Musser and Carleton [6] checklist, which is one of the most cited taxonomic references, listed 21 species in the genus *Lophuromys* and mainly followed Verheyen et al*.* [12] in recognizing seven of these species under an *L. aquilus* group based on craniodental affinities (*L. aquilus* [23], *L. brunneus* [24], *L. chrysopus* [19], *L. dieterleni* [25], *L. eisentrauti* [26], *L. verhageni* [12], and *L. zena* [27]). Six other species were considered as synonyms of *L. aquilus* by Musser and Carleton [6]: *L. cinereus* [28], *L. laticeps* [29], *L. major* [29], *L. margarettae* [30], *L. rita* [31], and *L. rubecula* [27]. However, Dieterlen [22] recently considered *L. aquilus, L. cinereus, L. laticeps, L. major, L. margarettae, L. rita,* and *L. rubecula* as morphotypes/synonyms of *L. flavopunctatus*. Yet, in the most recent checklists—Monadjem et al*.* [7], Denys et al*.* [5], and Burgin et al*.* [32]/Mammal Diversity Database [33]—virtually all species previously associated with the genus are considered as valid. This steady increase in newly recognized species suggests undescribed diversity in the genus *Lophuromys* and promotes debate over ‘species concepts,’ especially involving morphospecies, thus, demanding further taxonomic and biogeographic reevaluations.

The NONETHFLAVO members are among the most abundant small mammal fauna in forests of the Eastern Afromontane biodiversity hotspot south of the Ethiopian Highlands, including the Kenya Highlands, Albertine Rift montane forests, Tanzanian Highlands, and the Southern Rift montane forests [5, 7, 11, 15, 22, 34–36]. As such, they are a key ecological component in these biodiversity hotspots, serving both as prey to raptors and small carnivores and as predators of invertebrates [11, 37]. Moreover, the NONETHFLAVO members occur primarily in ecosystems characterized by limited variation in precipitation between seasons, making them ideal models for investigating how ecosystem-climate processes impact species ecological assembly, phylogeographic and evolutionary pathways. Altogether, they demand stable taxonomic accounts to guarantee accurate appraisal of taxonomic diversity and ecological roles which is vital to the accounting of the high faunal diversity recorded in their range for application in biodiversity conservation.

The current distribution of the genus *Lophuromys* reflects relatively well-structured phylogeographic patterns. The *L. sikapusi* group spans a pantropical African range, from western Guinea to western Kenya, the *Kivumys* group is restricted between the central Congo Basin and the Albertine Rift, and the *L. flavopunctatus* group is distributed primarily in eastern to central Africa [5, 7, 10, 11, 22]. Despite this remarkable geographic range, the spatiotemporal influence of geographical features and climatic oscillations on the historical biogeography and evolutionary radiation in the genus *Lophuromys* remains largely unknown [7]. Studies using larger genomic datasets, like Komarova et al*.* [16], have uncovered complex reticulate evolution and recurrent mitochondrial introgression among the ETHFLAVO members. This suggests other non-Ethiopian *Lophuromys* taxa might have undergone similar evolutionary pathways, illustrating that single-gene phylogenies, especially mitochondrial loci, should be interpreted with caution when utilized as the exclusive basis for taxonomic assignment. For the NONETHFLAVO members, even knowledge of mitochondrial DNA (mtDNA) diversity is limited. There is a necessity first to test the extent to which the mtDNA reflects taxonomic units and biogeographical trends across its distribution, and then to contrast it with nuclear data.

In this study, we evaluated the taxonomic limits and biogeographic patterns in the genus *Lophuromys* using a comprehensive mtDNA (*Cytochrome b*; *CYTB*) dataset. We then focused on the NONETHFLAVO members and complemented the *CYTB* alignment with *Cytochrome c oxidase I* (*COI*) and *Interphotoreceptor retinol-binding protein* (*IRBP*) and two morphometric datasets (geometric landmarks and linear measurements). The specific aims were (i) to elucidate the systematics of the NONETHFLAVO members in the context of their position in the genus *Lophuromys* and (ii) to elucidate the evolutionary and biogeographic history of the NONETHFLAVO members.

## Results

### Mitochondrial (*CYTB*) phylogeny of the genus *Lophuromys* and the definition of the *L. flavopunctatus* group

**Fig. 1:**
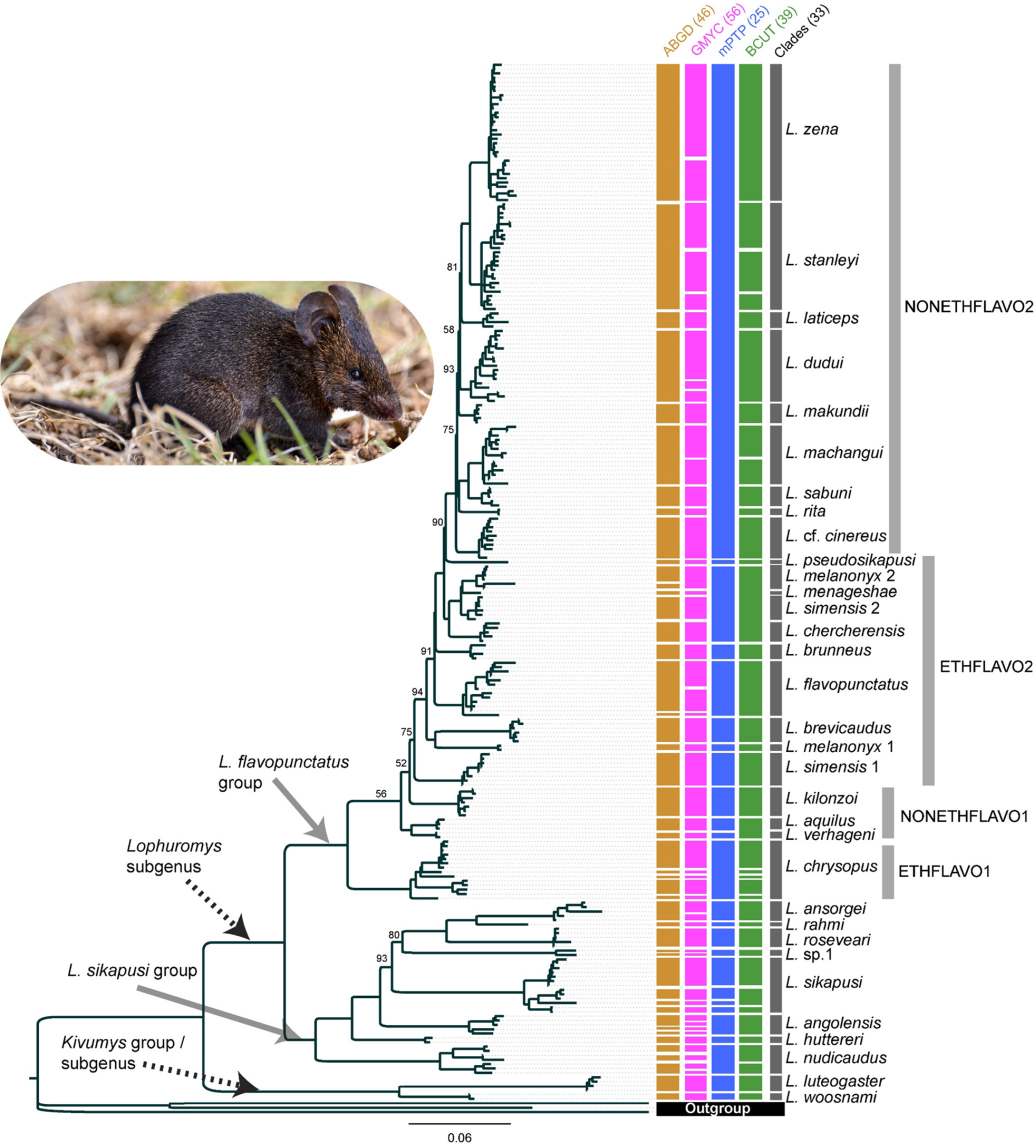
The phylogeny of the genus *Lophuromys* inferred from *Cytochrome b* gene in IQ-TREE using Maximum Likelihood phylogenetic inference. Values above branches represent bootstrap support values < 95 percent. The taxa labels ‘Clades’ represent the species identities of main clades resolved following operational taxonomic units (OTUs) suggested by the various species delimitation methods shown, with the corresponding number of OTUs in brackets. The inset image is used to illustrate the external body profile of *Lophuromys* rats (*L. brevicaudus* from the Bale Mountains, Ethiopia) and was provided by one of the authors (JB)

The genus-wide *CYTB* alignment produced congruent gene tree topologies for the Bayesian inference (BI) and maximum likelihood (ML) analyses (Fig. 1, Additional file 1: Fig. S1). In both trees, the genus *Lophuromys* bifurcated into two main groups that corresponded to the current subgeneric divisions—*Lophuromys* and *Kivumys* (Fig. 1, Additional file 1: Fig. S1). The *Lophuromys* branch split further into two groups, representing the *L. sikapusi* group and the *L. flavopunctatus* group (Fig. 1, Additional file 1: Fig. S1). In the *L. flavopunctatus* group, the non-Ethiopian samples (NONETHFLAVO1 and NONETHFLAVO2 in Fig. 1 and Additional file 1: Fig. S1) and samples from the Ethiopian Highlands (ETHFLAVO1 and ETHFLAVO2 in Fig. 1 and Additional file 1: Fig. S1) did not form separately monophyletic clades.

The major clades in the *Kivumys* group and *L. sikapusi* group corresponded to currently recognized species except for a single clade in the *sikapusi* group (*L.* sp.1 in Fig. 1 and Additional file 1: Fig. S1) and were assigned names based on the corresponding identifications in literature. These included two clades in the *Kivumys* group (*L. woosnami* and *L. luteogaster*) and eight clades in the *L. sikapusi* group (*L. sikapusi, L. nudicaudus, L. roseveari, L. ansorgei, L. huttereri, L. angolensis, L. rahmi,* and *L.* sp.1). Similarly, the major clades in ETHFLAVO1 and ETHFLAVO2 matched recently clarified taxonomies [15–17], from which names were extracted (Fig. 1, Additional file 1: Fig. S1).

**Fig. 2:**
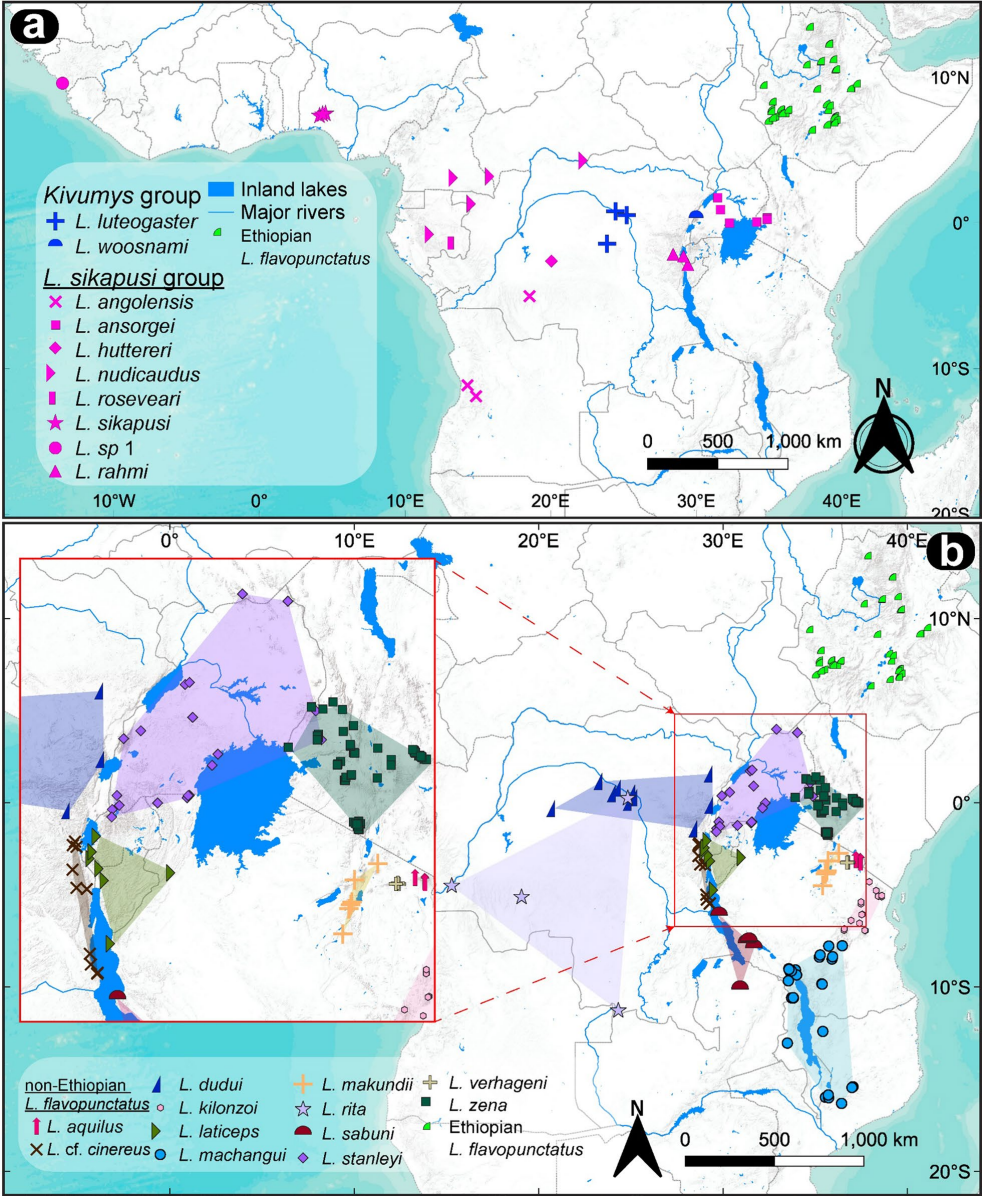
Topographic maps showing the geographical distribution of samples used in the study. **a** Sampling points of members of the *Kivumys* group*, L. sikapusi* group*,* and Ethiopian *L. flavopunctatus* group [see Komarova et al*.* [16] for the detailed per-species sampling points]; **b** Sampling points of non-Ethiopian *L. flavopunctatus* group members, with convex hulls indicating distribution extents (inset map zooms in on the red-outlined area for clarity), the corresponding type localities are shown in Additional file 1: Fig. S2

The three main species groups in the genus *Lophuromys* were well-supported (BS and PP > 0.95) and occupied relatively specific geographic areas (Fig. 2). The *L. flavopunctatus* group was distributed primarily in highland regions of east and east-central Africa, with the ETHFLAVO members being endemic to Ethiopia and the NONETHFLAVO members spanning a broader range over the Eastern Afromontane Highlands south of Ethiopia (Fig. 2). The *L. sikapusi* group traversed the Guinea-Congo forest belt, with a primarily west to central Africa range (Fig. 2), while the *Kivumys* group distribution was restricted between the Albertine Rift and the central Congo Basin (*L. luteogaster*), where it overlapped ranges with the *L. sikapusi* group and several NONETHFLAVO clades (Fig. 2).

Overall, between-group genetic distance (uncorrected *p*-distance) was highest in the *Kivumys* group versus *L. sikapusi* group (16.9%), the *Kivumys* group versus *L. flavopunctatus* group was comparably distant at 16.4%, and the *L. sikapusi* group versus *L. flavopunctatus* group were relatively less differentiated (11.5%).

### Mitochondrial phylogeny of the *L. flavopunctatus* group

Within the *L. flavopunctatus* group, 12 main clades were resolved from the NONETHFLAVO samples; three in the first subgroup—NONETHFLAVO1—and nine in the second subgroup—NONETHFLAVO2 (Fig. 1). The ETHFLAVO1 and ETHFLAVO2 subgroups were separated by an 8.74% genetic *p*-distance, slightly higher than the 5.8% *p*-distance that separated NONETHFLAVO1 and NONETHFLAVO2. Over-all, the *p-*distances between clades corresponding to the NONETHFLAVO (NONETHFLAVO1 and NONETHFLAVO2) were comparable, albeit averagely lower, compared to between ETHFLAVO clades (Table 1).

**Table 1:** Estimates of evolutionary divergence between and within clades in the *L. flavopunctatus* group

Clade	**1**	**2**	**3**	**4**	**5**	**6**	**7**	**8**	**9**	10	11	12	13	14	15	16	17	18	19	**20**	**21**	**22**	23
**1 *L. zena***	**0.9**	31.6	40.4	33.3	31.2	41.7	41.2	46.6	38.6	49.3	40.9	32.4	46.0	48.3	43.8	47.3	58.3	60.8	49.0	64.0	64.9	64.4	73.9
**2 *L. stanleyi***	2.9	**1.4**	36.6	30.0	29.8	40.5	41.6	48.6	34.7	44.4	35.4	32.9	43.2	45.1	46.3	46.5	54.2	60.9	48.3	66.7	65.2	66.7	70.1
**3 *L. laticeps***	3.8	3.4	**1.1**	28.8	27.8	36.3	40.3	47.7	39.5	46.6	37.3	32.3	42.1	47.0	43.6	45.0	59.4	65.3	47.0	63.8	63.4	61.8	77.4
**4 *L. dudui***	3.6	3.2	3.1	**1.6**	20.1	31.0	31.0	33.9	30.9	36.2	29.9	24.0	28.3	39.1	35.2	39.7	50.5	48.1	43.6	51.4	52.5	50.3	68.3
**5 *L. makundii***	3.4	3.2	3.0	2.4	**0.6**	27.5	28.1	31.9	28.2	36.2	30.6	25.7	31.3	36.7	32.7	37.0	53.9	46.2	38.7	43.1	44.3	41.3	65.5
**6 *L. machangui***	3.9	3.7	3.4	3.4	3.0	**1.3**	29.4	46.2	34.8	42.2	39.2	33.2	43.2	46.7	45.5	43.5	58.4	61.3	45.5	62.3	59.7	59.6	75.5
**7 *L. sabuni***	3.8	3.8	3.8	3.3	3.0	2.7	**0.6**	44.9	36.9	39.2	38.0	32.7	41.7	45.1	40.1	42.7	59.8	63.3	47.7	63.6	60.2	60.4	76.5
**8 *L. rita***	4.2	4.4	4.4	3.6	3.4	4.2	4.1	**0.2**	44.1	44.5	42.9	36.3	43.5	50.9	47.4	49.0	62.7	60.1	47.0	67.2	68.1	65.5	80.7
**9* L.* cf. *cinereus***	3.5	3.1	3.7	3.3	3.0	3.2	3.4	4.0	**0.9**	41.6	36.6	33.2	44.6	46.3	41.7	45.0	57.7	58.9	47.8	66.0	59.3	60.8	71.7
10 *L. pseudosikapusi*	5.7	5.1	5.4	4.5	4.5	4.8	4.5	5.0	4.7	**0.3**	37.7	34.5	40.1	42.8	44.0	44.8	53.7	54.7	46.4	57.7	52.3	49.2	76.6
11 *L. melanonyx* 2	4.6	3.9	4.2	3.7	3.8	4.4	4.2	4.7	4.1	4.8	**0.7**	17.2	21.4	35.9	36.5	42.8	50.8	50.3	45.0	52.4	47.8	47.7	69.1
12 *L. menageshae*	3.8	3.8	3.8	3.0	3.2	3.9	3.8	4.2	3.8	4.4	2.3	**0.2**	15.5	30.4	31.1	39.4	45.6	50.6	40.6	47.8	47.2	44.9	68.0
13 *L. simensis* 2	4.7	4.3	4.3	3.2	3.5	4.4	4.2	4.4	4.5	4.6	2.5	1.8	**0.6**	38.5	37.8	46.1	53.9	56.0	44.2	56.3	54.6	51.6	75.2
14 *L. chercherensis*	5.1	4.7	5.1	4.7	4.3	5.0	4.8	5.3	4.9	5.2	4.4	3.8	4.3	**0.8**	43.2	41.6	62.9	63.8	53.1	60.1	60.4	54.6	82.0
15 *L. brunneus*	4.8	5.1	4.8	4.3	3.9	5.0	4.4	5.2	4.5	5.4	4.6	4.0	4.3	5.1	**0.7**	40.2	53.0	50.8	39.5	46.5	46.5	47.3	67.5
16 *L. flavopunctatus*	5.4	5.3	5.2	5.0	4.6	4.9	4.9	5.5	5.1	5.7	5.5	5.1	5.3	5.1	5.0	**1.3**	52.6	55.8	47.4	51.4	48.6	48.7	70.6
17 *L. brevicaudus*	5.9	5.5	6.1	5.9	6.2	5.9	6.1	6.3	5.8	6.4	6.0	5.5	5.8	7.1	6.1	6.2	**0.5**	61.6	51.4	62.9	64.4	65.9	72.9
18 *L. melanonyx* 1	5.8	5.7	6.3	5.5	5.1	5.8	6.0	5.6	5.5	6.5	5.8	6.1	5.9	7.0	5.8	6.5	6.5	**0.3**	45.0	68.2	57.9	57.7	76.8
19 *L. simensis* 1	6.1	6.0	5.9	5.8	5.2	5.6	5.9	5.8	5.9	6.3	6.2	5.6	5.6	7.0	5.3	6.5	6.6	5.8	**0.9**	43.9	39.2	44.0	55.6
**20 *L. kilonzoi***	5.9	6.1	6.0	5.6	4.6	5.8	5.9	6.1	6.0	6.6	5.9	5.6	5.7	6.4	5.1	5.8	6.4	6.5	5.4	**1.0**	51.7	52.6	78.5
**21 *L. verhageni***	5.9	5.8	5.9	5.6	4.7	5.4	5.4	6.1	5.3	6.0	5.3	5.5	5.5	6.4	5.0	5.5	6.5	5.4	4.8	4.7	**0.2**	32.0	73.6
**22 *L. aquilus***	5.9	6.0	5.7	5.4	4.4	5.4	5.5	5.9	5.5	5.6	5.3	5.2	5.2	5.7	5.1	5.5	6.6	5.4	5.5	4.8	2.8	**0.4**	77.0
23 *L. chrysopus*	8.4	7.9	8.9	8.5	8.1	8.6	8.7	9.1	8.1	9.7	8.8	8.8	8.8	10.0	8.3	8.9	8.7	9.0	7.5	9.0	8.3	8.7	**2.0**

The number of base substitutions per site from averaging over all sequence pairs between groups are shown for within-clade (bold-font diagonal) and between-clade (upper matrix: actual average site differences, lower matrix: uncorrected *p*-distances) comparisons estimated in MEGA X [95]. The non-Ethiopian *L. flavopunctatus* members are highlighted in bold-font clade names

**Fig. 3:**
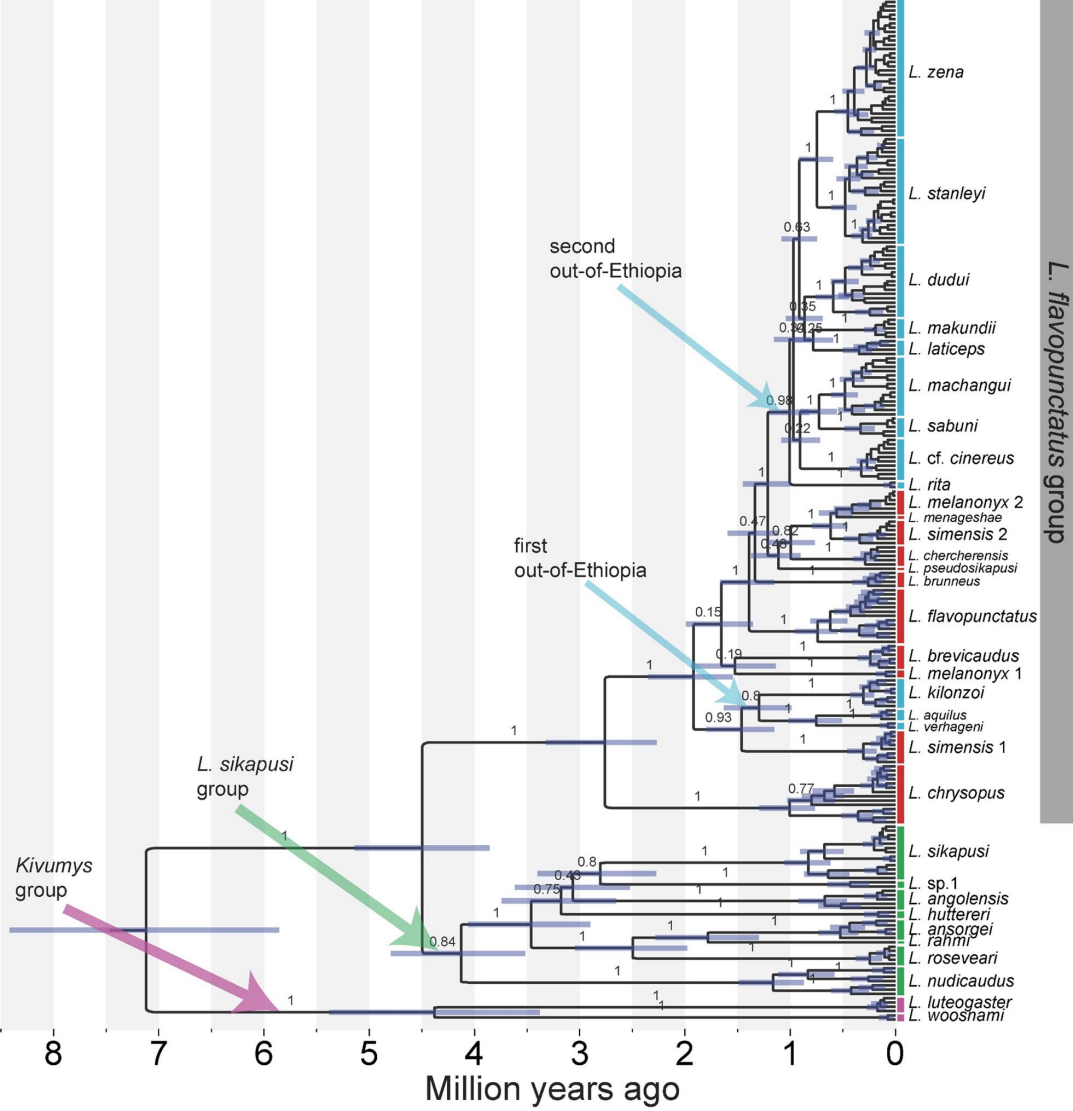
Time-calibrated maximum clade credibility tree showing the evolutionary relationships and divergence times in the genus *Lophuromys*. The tree was reconstructed based on *Cytochrome b* using secondary ‘most recent common ancestor’ calibrations. Branch labels show the posterior probability support values for main branches only. Node bars represent the highest posterior density interval of median ages. Bars delimit clade boundaries, and colors, including the matching arrow colors, indicate the species groups

The first NONETHFLAVO subgroup, NONETHFLAVO1, was comprised of three clades—*L. aquilus, L. verhageni,* and *L. kilonzoi* (Fig. 1). The *L. aquilus* clade was separated by 2.84% *p*-distance from the sister clade, *L. verhageni,* and 4.81% from *L. kilonzoi* (Table 1). The *L. verhageni* was separated by a 4.7% *p*-distance from *L. kilonzoi* clade, which was sister to the *L. aquilus* + *L. verhageni* clade (Figs. 1 and 3) and more diverse than both (Table 1).

The second NONETHFLAVO subgroup, NONETHFLAVO2, contained nine distinct clades—*L. machangui, L. sabuni, L. makundii, L. dudui, L. rita, L.* cf. *cinereus, L. laticeps, L. stanleyi,* and *L. zena* (Figs. 1 and 3). The phylogenetic relationships and divergence times between clades are shown in Figs. 1 and 3, their respective geographic ranges in Fig. 2 and Additional file 1: Fig. S2, and their evolutionary diversity in Table 1.

### Concatenated mitochondrial and nuclear phylogeny of the non-Ethiopian *L. flavopunctatus* members

The concatenated mitochondrial tree of the NONETHFLAVO was generally congruent to the genus-wide *CYTB* topology, with minor differences in sister relationships between clades (Additional file 1: Fig. S3). The NONETHFLAVO1 subgroup was distinct from NONETHFLAVO2, each separately monophyletic (Additional file 1: Fig. S3). In the NONETHFLAVO1 subgroup, notable differences with the *CYTB* topology included the paraphyly of *L. zena* + *L. stanleyi* clade, which contrasted the monophyly in the *CYTB* tree. The *L. zena* and *L. stanleyi* clades were also positioned at the root of NONETHFLAVO2, unlike in the *CYTB* tree. Except for *L. laticeps* and *L. rita,* which were not successfully sequenced for *COI* and therefore not included in the concatenated mitochondrial analysis*,* the rest of the NONETHFLAVO clades maintained corresponding topologies to the *CYTB* tree (Additional file 1: Fig. S3). On the other hand, the nuclear (*IRBP*) phylogeny did not correspond to the *CYTB* or concatenated mitochondrial tree, with most clades included in polytomies (Additional file 1: Fig. S4). In the NONETHFLAVO1 subgroup, *L. aquilus* merged with *verhageni* in monophyly while the *L. kilonzoi* samples remained monophyletic but not sister to the *L. aquilus* + *L. verhageni* (Additional file 1: Fig. S4).

### Mitochondrial species delimitation, genetic distances, and networks

Each of the four delimitation methods produced an incongruous number and topology of splitting OTUs based on the genus-wide *CYTB* trees (Fig. 1). The mPTP identified 25 OTUs which differed from the 39 identified by BCUT, 46 identified by ABGD, and 56 identified by GMYC (Fig. 1). The *L. sikapusi* and *L. chrysopus* clades were consistently split into at least three OTUs across the methods, except in mPTP (Fig. 1). Several other clades were split as multiple OTUs by at least one of the delimitation methods, including those of NONETHFLAVO2 [*L. zena, L. stanleyi, L. dudui,* and *L. machangui*] (Fig. 1). Based on currently recognized species in literature and the haplotype networks, we resolved the 25–56 delimited OTUs to represent 33 clades. Of these, there were two clades in the *Kivumys* group, eight in the *L. sikapusi* group, and 23 in the *L. flavopunctatus* group [12 clades corresponding to the NONETHFLAVO and 11 clades corresponding to the ETHFLAVO (Fig. 1)].

**Fig. 4:**
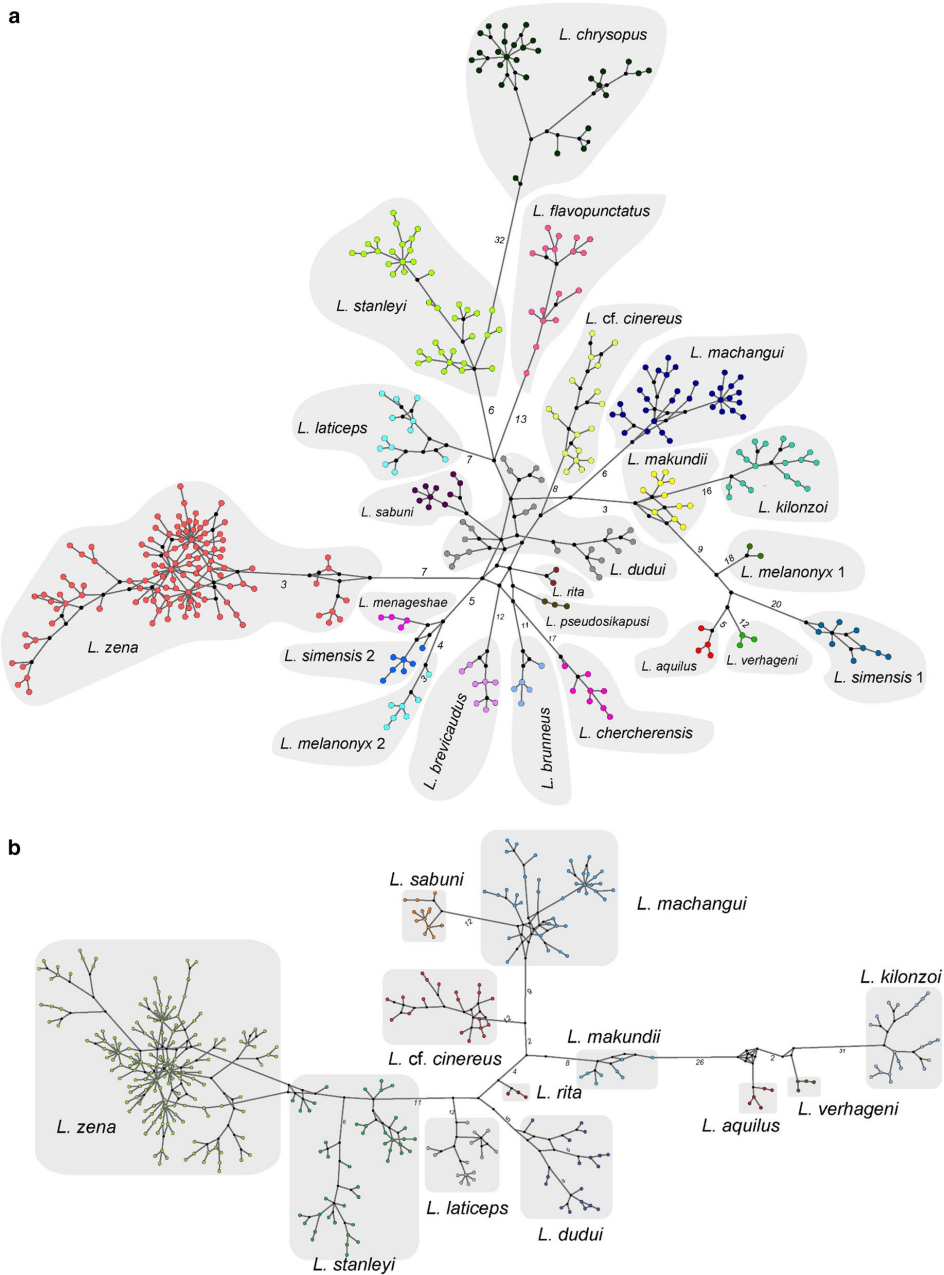
Haplotype network structure in the *L. flavopunctatus* group inferred from *Cytochrome b* using the Median Joining Network algorithm in PopART [117]. The networks are illustrated separately for the *L. flavopunctatus* group (**a**) and the non-Ethiopian *L. flavopunctatus* members (**b**). The number of base substitutions between haplotypes are shown as numbers for some of the main branches. The node sizes are fixed and do not correspond to the haplotype frequency (number of samples per haplotype) and branch lengths are relative but not proportional to the number of mutations between haplotypes

The evolutionary diversity (uncorrected *p*-distance) within the NONETHFLAVO clades (0.2–1.6%) was systematically lower than between-clade diversity (2.39–6.14%) (Table 1). The haplotype networks of the *L. flavopunctatus* group (combined ETHFLAVO and NONETHFLAVO) depicted composite genealogical relationships between clades that were not apparent in the phylogenetic trees, but altogether suggested a common evolutionary origin (Fig. 4). The more broadly sampled clades such as *L. zena*, *L. stanleyi*, and *L. machangui* had more haplotypes than those sampled from fewer localities such as *L. aquilus* and *L. verhageni* (Fig. 4). These broadly sampled clades also revealed that haplotype networks were only slightly influenced by sampling coverage, such that, within a clade, different localities were not uniquely systematically clustered (Additional file 1: Fig. S5).

### Divergence dating—time-calibrated trees

The *CYTB* divergence time estimates and phylogenetic associations between clades in the genus *Lophuromys* are presented in Fig. 3. Although deep divergences were well supported (PP > 0.95), most of the recent splits had low posterior support [PP < 0.95]. Divergence within the genus *Lophuromys* commenced ca. 7.12 Mya (HPDI: 5.86–8.42), resulting in the split of the genus into the two subgenera—*Kivumys* and *Lophuromys*. In the *Kivumys* subgenus, *L. luteogaster* and *L. woosnami* diverged ca. 4.38 Mya (HPDI: 3.38–5.38 Mya) while in the *Lophuromys* subgenus, the *L. sikapusi* and *L. flavopunctatus* groups diverged ca. 4.5 Mya (HPDI: 3.85–5.14). The earliest divergence in the *L. sikapusi* group occurred ca. 4.13 Mya (HPDI: 3.52–3.05) when *L. nudicaudus* split from the ancestor the rest of the group, within which divergences between ca. 2.49 Mya to ca. 1.78 Mya resulted in seven clades (Fig. 3). Internal divergences within the *L. flavopunctatus* group were more recent than in the *Kivumys* group and *sikapusi* group; with the oldest lineage, *L. chrysopus*, appearing ca. 2.76 Mya (HPDI: 2.27–3.32) but all other species appearing after the last divergence in the *L. sikapusi* group (Fig. 3). The ancestor of NONETHFLAVO1 diverged ca. 0.91 (HPDI: 0.69–1.04) Mya from *L. simensis* 1 while NONETHFLAVO2 diverged ca. 0.7 (HPDI: 0.55–0.86) Mya from *L. pseudosikapusi*. Internal divergences within NONETHFLAVO1 ca. 0.45–0.79 Mya led to three clades (*L. aquilus, L. verhageni,* and *L. kilonzoi*). Divergences within NONETHFLAVO2 ca. 0.41–0.61 Mya led to nine clades (*L. makundii, L. stanleyi, L. rita, L. zena, L. laticeps, L. dudui, L.* cf. *cinereus, L. machangui,* and *L. sabuni*)—Fig. 3.

### Historical biogeography of the genus *Lophuromys*

**Fig. 5:**
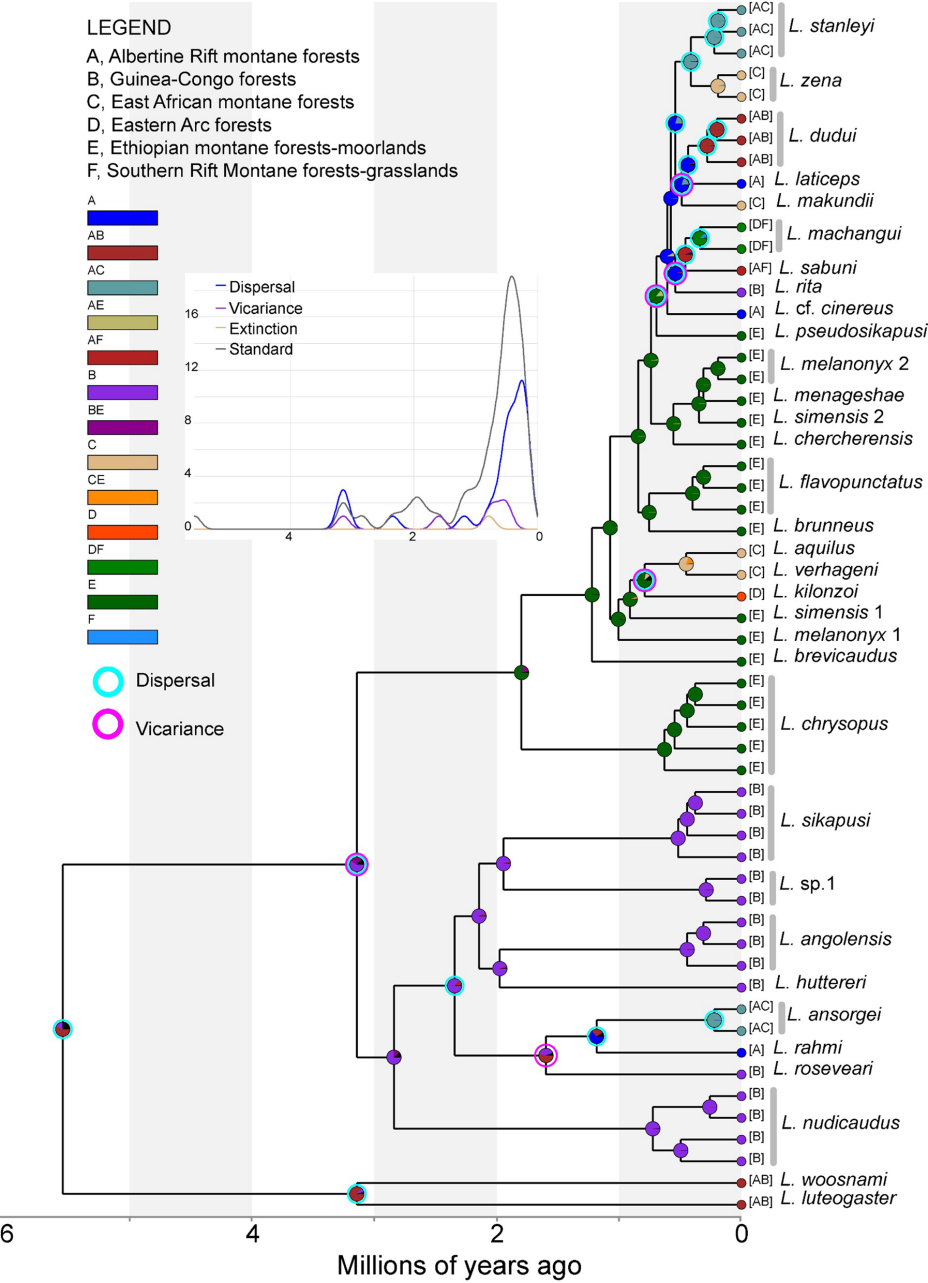
The historical ancestral areas and biogeography of the genus *Lophuromys.* The node shapes illustrate the suggested historical range at divergence, marked as color proportions of the biogeographic ecoregions in the legend. The suggested vicariance and dispersal events are also shown as node shapes. The inset graph shows the frequency of various ancestral origins (y-axis) against divergence time (x-axis)

Divergence within the genus *Lophuromys* likely originated in the Guinea-Congo/Albertine Rift forests, from where several dispersal events (28 dispersals versus six vicariance events) led to the colonization of current ranges (Fig. 5). These dispersals mostly occurred within ecoregions (mainly in the Guinea-Congo and Ethiopian Highlands forests) than between ecoregions (Fig. 5). The divergence in the *Kivumys* group likely originated in the same area as the genus*,* while in the *Lophuromys* subgenus, the Guinea-Congo forests formed the ancestral range, after which the *L. sikapusi* group remained in the Guinea-Congo forests while the *L. flavopunctatus* group dispersed to the Ethiopian Highlands. From the Ethiopian Highlands, the NONETHFLAVO species colonized current ranges over two southward dispersal events (Fig. 5). The first dispersal was by the NONETHFLAVO1 ancestor to the East African montane and Eastern Arc forests after which vicariance caused consequent divergences (Fig. 5). The NONETHFLAVO2 ancestor later dispersed to the Albertine Rift forests, from where both dispersal and vicariance events resulted in the colonization of the Congolian forests, East African montane forests, Eastern Arc forests, and the Southern Rift Montane forests (Fig. 5).

### Morphometric analysis of the non-Ethiopian *L. flavopunctatus* members

**Fig. 6:**
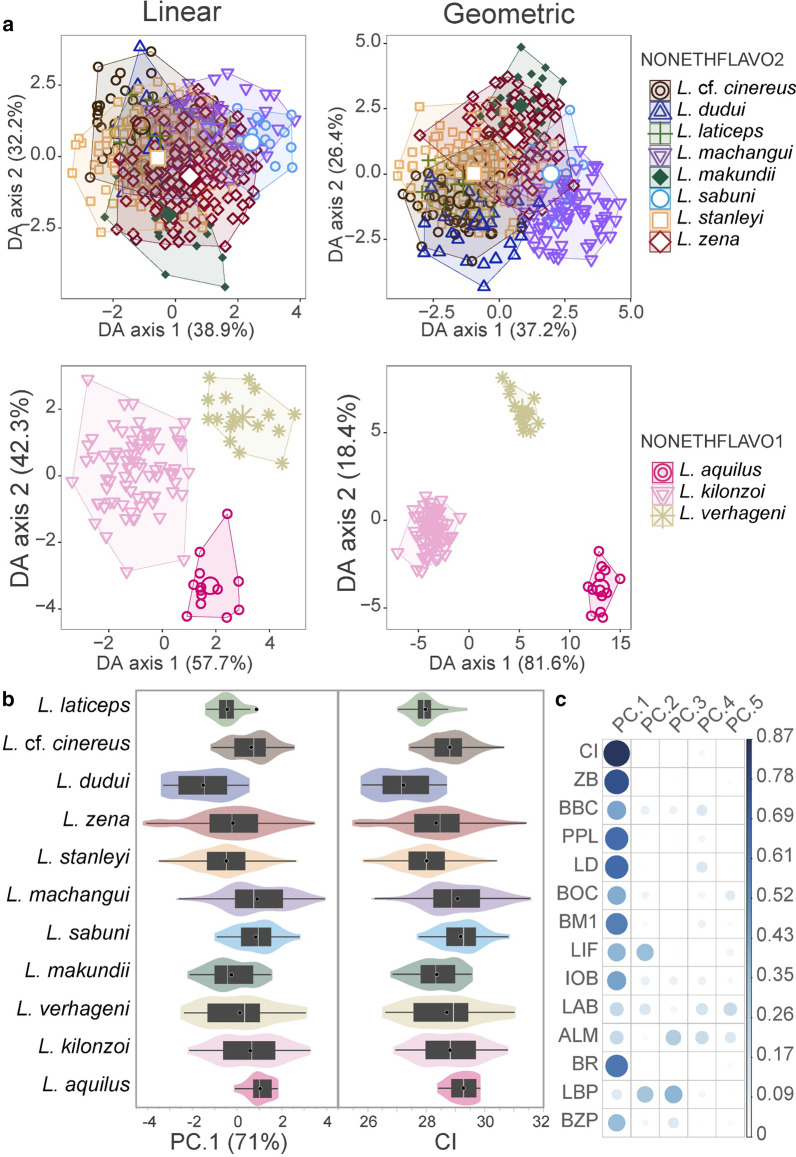
Craniodental variation between the non-Ethiopian *L. flavopunctatus* members. The scatterplots (**a**) indicate discriminant function analysis of linear and geometric morphometric characters with the x-axis and y-axis showing the percentage variance accounted for by the first and second discriminant scores, respectively. The plots are partitioned based on the two subgroups of the non-Ethiopian *L. flavopunctatus* members. The box/violin plots (**b**) show how the condyle-incisive skull length (CI) and the first axis (PC1) of a principal component analysis (PCA) using the 14 linear measurements compare between clades. The contributions of each measurement to the PCA loadings on the first five axes (PC.1–PC.5) are shown in **c**. The violin breadth illustrates the spread of individual samples around the mean (white outlined black dot) and median (transparent line dividing boxes)

Overall, *L. dudui* had the smallest skull, while the *L. aquilus* skulls were the largest (Fig. 6, Additional file 2: Table S1). The morphospace of the combined clades following linear (DA^LIN^) and geometric (DA^GEO^) discriminant tests overlapped randomly with no evident systematic pattern delimiting the *CYTB* clades. Therefore, we partitioned the datasets into two groups around the two phylogenetic subgroups; NONETHFLAVO1 (*L. aquilus*, *L. verhageni*, *L. kilonzoi*) and NONETHFLAVO2 (*L. sabuni, L. makundii,* and *L. machangui, L. stanleyi*, *L. dudui*, *L. laticeps, L.* cf. *cinereus*, and *L. zena*).

The DA^LIN^ and DA^GEO^ classification results were consistent over-all, however, most clades were more correctly classified (more distinguishable) by DA^GEO^, especially in NONETHFLAVO2 (Fig. 6, Additional file 1: Fig. S7). Between-clade differences between the two skull datasets were not unidirectional, with DA^GEO^ achieving lower correct classification than DA^LIN^ in NONETHFLAVO1 but not in NONETHFLAVO2 (Table 2). In NONETHFLAVO1, all three clades were distinct, with DA correctly classifying > 85% of each clade into the respective given group (Table 2). The *L. aquilus* and *L. verhageni* were the most correctly classified by either DA^LIN^ or DA^GEO^ (Table 2, Fig. 6). The *L. verhageni* skulls were smaller than the adjacent *L. aquilus* or *L. kilonzoi* (Additional file 2: Table S1), but with *L. verhageni* and *L. aquilus* skulls more closely related to each other more than to *L. kilonzoi* (Fig. 6, Additional file 1: Fig. S7, Table 2).

**Table 2:** The classification of the non-Ethiopian *L. flavopunctatus* members based on discriminant analysis of linear and geometric craniodental characters

(a)					
	1	2	3	*N*	*σ*
Linear					
1 *L. aquilus*	**92.3**	0	7.7	13	95.2
2 *L. kilonzoi*	2.7	**94.6**	2.7	74	
3 *L. verhageni*	0.0	0.0	**100**	17	
Geometric					
1 *L. aquilus*	**84.6**	0	15.4	13	87.4
2 *L. kilonzoi*	0	**88.1**	11.9	67	
3 *L. verhageni*	13.3	0	**86.7**	15	

(b)										
	*1*	*2*	*3*	*4*	*5*	*6*	*7*	*8*	*N*	*σ*
Linear										
1 *L.* cf. *cinereus*	**57.4**	4.9	16.4	4.9	4.9	1.6	8.2	1.6	61	44.6
2 *L. dudui*	13.8	**55.2**	20.7	0	0	0	6.9	3.4	29	
3 *L. laticeps*	23.1	3.8	**38.5**	7.7	0	3.8	19.2	3.8	26	
4 *L. machangui*	5.9	4.7	4.7	**60.0**	0	10.6	5.9	8.2	85	
5 *L. makundii*	3.3	0	0	0.0	**83.3**	0	6.7	6.7	30	
6 *L. sabuni*	0	0	0	13.6	0	**81.8**	0	4.5	22	
7 *L. stanleyi*	13.7	10.9	12.8	6.6	7.6	1.9	**35.5**	10.9	211	
8 *L. zena*	3.2	10.2	7.6	13.4	20.4	5.7	9.6	**29.9**	157	
Geometric										
1 *L.* cf. *cinereus*	**47.4**	8.8	10.5	5.3	5.3	0	19.3	3.5	57	59.4
2 *L. dudui*	24.1	**51.7**	6.9	0	0	3.4	13.8	0	29	
3 *L. laticeps*	14.3	9.5	**42.9**	0	0	4.8	19	9.5	21	
4 *L. machangui*	2.5	1.3	0	**79.7**	1.3	8.9	3.8	2.5	79	
5 *L. makundii*	0	3.6	0	0	**85.7**	0	0	10.7	28	
6 *L. sabuni*	0	5.3	0	15.8	0	**63.2**	5.3	10.5	19	
7 *L. stanleyi*	13.1	6	9	2.5	3	1.5	**53.8**	11.1	199	
8 *L. zena*	3.7	3.7	3.7	6.5	7.5	5.6	10.3	**58.9**	107	

The results are partitioned by the two subclades; NONETHFLAVO1 in **a** and NONETHFLAVO2 in **b**. Values represent the cross-validated (leave-one-out bootstrapping) percentage success by which samples were predicted into the corresponding species group. The diagonal values in bold fonts indicate the percentage success by which samples were predicted into their groups which correspond to the *Cytochrome b* clades in Fig. 1. The classification of the combined clades is shown in Additional file 1: Fig. S7

*σ* = overall classification success, *N* = number of samples

In NONETHFLAVO2, the morphospace of *L. zena* and *L. stanleyi* markedly overlapped in DA^LIN^ and DA^GEO^, between themselves and with several other clades, mainly *L.* cf. *cinereus, L. dudui,* and *L. laticeps* (Fig. 6, Table 2, Additional file 1: Fig. S7). The *L. laticeps* skulls were highly indistinguishable from other clades, being least correctly classified in the NONETHFLAVO2 subgroup and the combined pool of all clades.

The range-restricted clades, such as *L. verhageni, L. aquilus,* and *L. makundii*, were less ambiguously delimited and highly correctly classified by DA^LIN^ and DA^GEO^ (Fig. 6, Table 2, Additional file 1: Fig. S7). In contrast, more broadly sampled clades such as *L. zena* and *L. stanleyi* were less distinctly discriminated against from other clades (Fig. 6, Table 2). Permutational multivariate analysis of variance (PerMANOVA) showed overall differences between clades were significant (Linear dataset: *F* = 15.13, *p* = 0.0001, Geometric dataset: *F* = 10.1, *p* = 0.0001), although pairwise post hoc tests showed differences between some clade pairs were nonsignificant (Additional file 2: Table S2).

## Discussion

### Phylogenetic relationships within the genus *Lophuromys*

The deeper phylogenetic relations in the genus *Lophuromys,* including the validity of the subgeneric divisions (*Lophuromys* and *Kivumys*) and older lineages (*Kivumys* group and *L. sikapusi* group), and their respective monophyly, have been relatively uncontested in recent checklists [5, 6, 22]. In contrast, species accounts in the ‘speckled pelage’ groups, the *L. flavopunctatus* group, combining the Ethiopian endemics—ETHFLAVO) and the non-Ethiopian ones—NONETHFLAVO*,* have changed substantially recently. In consensus, our genus-wide phylogenetic inference based on the *CYTB* gene supports the deep divergence of the genus *Lophuromys* into three distinct deeply-diverged groups that correspond with the widely recognized species groupings; (i) *Kivumys* group (*Kivumys* subgenus), (ii) *L. sikapusi* group (*Lophuromys* subgenus), and (iii) *L. flavopunctatus* group (*Lophuromys* subgenus). The two *Lophuromys* groups—*L. sikapusi* group and *L. flavopunctatus* group—are separated by a much lower mtDNA divergence (*p*-distance) compared to the almost equidistant *p*-distance separating them from the *Kivumys* group.

Within the *Kivumys* group (subgenus *Kivumys*), high *CYTB* differentiation (13.39% *p*-distance) between the two species represented in our dataset—*L. woosnami* and *L. luteogaster*—clearly delimitate them as distinct lineages. Together with *L. medicaudatus,* whose *CYTB* sequences were not included in the study, all three species in the *Kivumys* subgenus have been recorded from overlapping ranges, i.e., in the northeastern and eastern DRC forests and bordering montane forests of the Albertine Rift, with *L. woosnami* extending into western Burundi, Rwanda, and Uganda [5, 6, 22, 35, 38]. A thorough investigation of niche partitioning and other ecomorphological strategies inherent in gene flow and adaptive genetic divergence within the *Kivumys* subgenus is necessary to clear up their evolutionary history. Such a study would also illuminate the precise nature and limits of their ranges (whether sympatric, syntopic, or parapatric).

The eight clades in the *L. sikapusi* group correspond to seven described species (*L. angolensis, L. ansorgei, L. huttereri, L. nudicaudus, L. rahmi, L. roseveari,* and *L. sikapusi*) and an unidentified taxon (*L.* sp.1 in Fig. 1). The *L.* sp.1 clade is separated by 10.47–13.85% *p*-distance from all other species in the *L. sikapusi* group and forms a sister relationship with *L. sikapusi* (separated by 11.42% *p*-distance). This clade represents a potentially undescribed species likely conspecific to the undescribed taxa that Denys et al*.* [39] considered tentatively new in the *L. sikapusi* group.

The assignment of ETHFLAVO clades to corresponding species is a nontrivial task due to the recently clarified taxonomic accounts of the Ethiopian *Lophuromys* [15–17]. For example, the pairs of highly divergent *CYTB* clades of *L. simensis* (*L. simensis* 1 and *L. simensis* 2) and *L. melanonyx* (*L. melanonyx* 1 and *L. melanonyx* 2) comprise the multiple haplogroups within the same species due to past mtDNA introgression events. Such introgressions have also been confirmed in *L. brunneus,* of which we only sampled one haplogroup. Nuclear genomic data support the recognition of these 12 mtDNA lineages as nine species (*L. chrysopus, L. melanonyx, L. simensis, L. flavopunctatus, L. brunneus, L. pseudosikapusi, L. menageshae, L. chercherensis,* and *L. brevicaudus*) which differ by karyotypes, morphology, and preferred elevation, i.e., types of ecosystems [13, 15, 16]. Mitochondrial introgression, apparently common in the Ethiopian *L. flavopunctatus* endemics, was not detected in the rest of the genus, although it should be noted that nuclear genetic data are relatively scarce outside the Ethiopian *flavopunctatus* members*.*

Among the NONETHFLAVO, the three clades, *L. aquilus, L. verhageni,* and *L. kilonzoi*, forming a distinct subgroup (NONETHFLAVO1) phylogenetically isolated from the second subgroup, NONETHFLAVO2 (*L.* cf. *cinereus, L. dudui, L. laticeps, L. machangui, L. makundii, L. rita, L. sabuni, L. stanleyi,* and *L. zena*)—Figs. 1 and 3—reiterates that the Ethiopian and non-Ethiopian *Lophuromys* are deeply nested phylogenetically as a monophyletic ‘*L. flavopunctatus* group’. These findings also agree with previous conjectures that the NONETHFLAVO colonized the current ranges following dispersals out of Ethiopia [13, 14].

The utilization of pelage coloration to resolve the systematic grouping of species is rather debatable in the genus *Lophuromys*. Our *CYTB* tree showed all unspeckled-pelage taxa clustered in the *L. sikapusi* group (*L. angolensis, L. ansorgei, L. huttereri, L. nudicaudus, L. rahmi, L. roseveari, L. sikapusi,* and *L.* sp.1), well distinct from the speckled-pelage *L. flavopunctatus* group. From an ecomorphological outlook, the craniodental relationship between *L. dieterleni*, *L. eisentrauti* and the *L. flavopunctatus* group [25, 26] that informed their inclusion in the *L. flavopunctatus* group [6] (citing Verheyen et al*.* [25]) might simply be signals of convergent adaptive responses to local environments [40, 41], but taxonomically uninformative without genetic evidence. Then again, the genetic and craniodental affinity of the unspeckled *L. pseudosikapusi* to the Ethiopian endemics [13] confounds further the overall phylogenetic relationships within and between the speckled-pelage (*L. flavopunctatus* group) and unspeckled-pelage (*L. sikapusi* group) species. More genetic studies are needed to reconcile morphological with phylogenetic associations in the genus *Lophuromys*.

### Species divergence and biogeography

The nested phylogenetic and genealogical relationships between the ETHFLAVO and NONETHFLAVO (Figs. 1, 3 and 4) conform generally to evolutionary processes speculated previously [13, 14]. While our findings support the Ethiopian highlands as the cradle of the speckled-pelage *Lophuromys*, the precise nature of their evolutionary radiation, including processes characterizing the observed differentiation between clades remains a matter for speculation, mainly owing to the strong effect of mtDNA on the inferred relationships. In any case, it is currently not possible to ascertain whether long-distance dispersal and/or montane-forest bridges promoted the divergence and dispersal of NONETHFLAVO members out of the Ethiopian Highlands. Dispersal along a north–south axis, i.e., out of Ethiopia to southern Afromontane Highlands is relatively like that of other montane-adapted rodents [42–44] and attributed to montane forest expansion during Pliocene–Pleistocene interglacials.

The timing of the NONETHFLAVO1 and NONETHFLAVO2 out-of-Ethiopia dispersals, albeit based on a single mitochondrial locus, coincide with the repeated expansion and contraction/isolation of montane forests and their faunal assemblages during the humid intervals of Pleistocene glacial-interglacial cycles [17, 45–48]. Within the *L. flavopunctatus* group*,* these events likely connected the southern Ethiopian Highlands with Albertine Rift montane forests, and Kenyan and Tanzanian Highlands across the currently arid Turkana depression [43, 45, 49–51]. The *L. flavopunctatus* group is primarily restricted to humid/wet habitats which are currently confined to montane areas in East Africa. These species could only have dispersed when the East Africa Highlands were connected with similarly suitable habitats. The first out-of-Ethiopia dispersal by the NONETHFLAVO1 ancestor and consequent range retention in the northern EAMs concur with their prolonged stability that preceded the formation of most of the Kenya Highlands, Tanzanian Highlands, and Albertine Rift montane forests. The split and dispersal of the *L. aquilus* + *L. verhageni* clade from *L. kilonzoi*, the consequent split of *L. aquilus* from *L. verhageni,* and the appearance of several clades in the NONETHFLAVO2 subgroup, all happened in the mid-late Pleistocene. This coincides with wet climate periods that made it possible to cross currently dry valleys such as those isolating Mt. Kilimanjaro and Mt. Meru and the Turkana depression in northern Kenya and southern Ethiopia [44, 52]. The absence of genetic evidence of this first dispersal in East African highlands such as the Kenyan Highlands, suggests these mountains served as ‘stepping-stones’. We can presume that the ‘first colonizers’—NONETHFLAVO1—were replaced by the ‘second colonizers’—NONETHFLAVO2, such as *L. zena* in the Kenyan highlands, which were more successful. Whether or not the second colonizers hybridized with the first ones remains unclear from mtDNA, and genomic analyses should be applied to investigate this possibility.

The Albertine Rift Valley is a crucial biogeographical feature in the radiation of the NONETHFLAVO and is likely an active barrier to gene flow on either side. The four distinct clades whose ranges are separated by the Albertine Rift (*L. dudui, L. stanleyi, L. *cf.* cinereus*, and *L. laticeps*) suggest they are not able to cross and have not experienced gene flow since their divergence. While *L. stanleyi* occurs widely eastward of the Rwenzori Mountains, it does not extend west of the mountains, whereas the range of *L. dudui* begins in Virunga National Park, and only extends westwards. The Albertine Rift might have been a barrier to *L. stanleyi*’s westward dispersal and *L. dudui*’s eastward dispersal. This hypothesis is also consistent with the occurrence of *L. laticeps* on the eastern and *L.* cf.* cinereus* on the opposite western side of the Albertine Rift around Lake Kivu and Lake Tanganyika, with either presumptively unable to cross. Notably, *L. sabuni*, which is the only clade whose occurrence spans both flanks of the Albertine Rift, appear to have dispersed between the Rukwa Rift and Lake Tanganyika and then southwards to Chishimba Falls (Northern Zambia), where it was recently recorded (Sabuni et al*.* [53]. Other forest rodents have ranges that span the Albertine Rift, unlike observed here for *L. dudui, L. stanleyi, L.* cf.* cinereus*, and *L. laticeps*; for instance, *Malacomys longipes* [54] and *Praomys jacksoni* [55] occur on both sides of the Albertine Rift Valley.

### Morphological variation within the non-Ethiopian *flavopunctatus* members

Most species in the NONETHFLAVO have overlapping craniodental characters in morphospace, making our large dataset of linear measurements and geometric landmarks unreliable as the exclusive evidence to infer species limits. For instance, the range of skull morphology of *L. stanleyi* and *L. zena* (both linear and geometric) significantly resembles the skull forms of all other clades in the NONETHFLAVO, except *L. verhageni* and *L. aquilus,* which unambiguously cluster and have the least overlap with any other species in the group. The *L. stanleyi* and *L. zena* clades exemplify a typical systematic problem in the NONETHFLAVO, where morphological evidence cannot classify samples to meaningful species units using taxonomically informative characters. Accounting for phenetic variation in the NONETHFLAVO, beyond their common ancestry, requires more comprehensive genomic analyses to disentangle the underlying ecomorphological processes among species occurring in similar habitats. Without such genomic evidence, the taxonomic accounts of several clades are best not considered reliably resolved when based on linear or geometric morphometrics only.

Divergence dates and biogeographic patterns in the NONETHFLAVO suggest that the drivers of craniodental variation fit multiple non-exclusive hypotheses associated with the correlation of ecomorphological divergence with speciation [56]. The relatively recent divergence of most of the clades suggests ecologically-mediated adaptive evolution might not be predominant speciation drivers between congeners in sympatry [57–59]. Except for *L. aquilus* and *L. verhageni* which are restricted to single mountain ecosystems, all the NONETHFLAVO species appear to have non-specialized niches as they are not restricted to high montane habitats. They are, thus, more likely to exhibit non-specialized morphological traits that are taxonomically uninformative [60]. The treatment of the *L. flavopunctatus* group by Verheyen et al*.* [12] and Verheyen et al*.* [14] highlights the use of craniodental and external morphology data to recognize populations as unique species with minimal use of genetic data. However, inter/intraspecific taxonomic delimitation among rodents often have fewer taxonomically informative stable morphological states, possibly due to nonadaptive and or rapid adaptive radiations [61]. These influences might hinder a replicable definition of taxon-specific phenotypic traits [62–64], leading to the subjective interpretation of valid species. While our geometric landmarks appears generally more sensitive at detecting variabilities between clades compared to linear measurements, just like in other cases [65], over-all, both datasets produced virtually similar results.

### Taxonomic assessment of the non-Ethiopian *flavopunctatus* members

While most of the OTUs recovered in the *L. flavopunctatus* group represent species currently named*,* the between-clade genetic distances and *CYTB* incongruence with morphometric and *IRBP* gene results raise more taxonomic questions than resolutions. For instance, only a few mutations at *CYTB* separated *L. aquilus* from *L*. *verhageni* (2.8% *p*-distance) and *L*. *stanleyi* from *L*. *zena* (2.9% *p*-distance) which is among the closest between-species *CYTB* divergences in the *L. flavopunctatus* group (Table 1). While such low sequence divergence between these sister clades indicates a recent separation of gene pools (at least at mtDNA), it nonetheless, raises concerns about the species’ taxonomic validity, suggesting the possibility of synonymizing them in future taxonomic revisions without more genetic support, especially since no clear diagnostic morphological differences delimit them. Moreover, the *IRBP* failure to delimitate several distinct mtDNA clades in the NONETHFLAVO might relate to its slow mutation rate which makes it unable to resolve deeper and or short branches between rodent species [66, 67]. Nevertheless, future taxonomic reassessments of the genus *Lophuromys* should utilize more comprehensive genomic analysis (such as multiple nuclear loci through ddRAD sequencing and multispecies coalescent species delimitation models such as STACEY and BPP delimitation) for more informative inference of phylogenetic associations. Such genomic evidence would also elucidate the level of distinctiveness between close relatives that are allopatric such as *L. aquilus* and *L. verhageni* and the level/absence of gene flow between parapatric ones such as *L. stanleyi* and *L. zena* [16].

The high genetic diversity within lineages such as *L. chrysopus* (Table 1), for instance, resulted in the delimited OTUs within them being as distinguishable pairwise as several clades in the NONETHFLAVO. It appears that taxonomic classifications of *Lophuromys* species that is not based on extensive nuclear evidence*,* should be regarded inconclusive (i.e., within the *Kivumys* group, *L. sikapusi* group, and NONETHFLAVO).

#### The NONETHFLAVO1 subgroup—*L. aquilus, L. verhageni,* and *L. kilonzoi*

The samples from Mt. Kilimanjaro, Mt. Meru, and northeastern Tanzanian Eastern Arc Mountains form distinct monophyletic lineages, representing species currently recognized as valid. *L. aquilus* was described by True [23] from Mt. Kilimanjaro and confirmed by Verheyen et al*.* [14] to be the only *Lophuromys* along the entire elevation gradient. *Lophuromys verhageni* was described by Verheyen et al*.* [12] as an endemic of Mt Meru, while *L. kilonzoi* was described by Verheyen et al*.* [14] from the Magamba, East Usambara. Perhaps because of fewer informative sites in shorter sequences, *L. aquilus, L. verhageni,* and *L. kilonzoi* had a different phylogenetic topology in Verheyen et al*.* [14]. Our expanded *CYTB* sampling supports the three species are minimally differentiated, forming a sister clade to one of the haplogroups of *L. simensis*. The current *CYTB* phylogeny, therefore, provides a clearer picture of the phylogenetic relationship between *L. aquilus, L. verhageni,* and *L. kilonzoi* and their position in the genus. Historical biogeographical reconstruction suggested that the colonization and divergence of *L. aquilus* and *L. verhageni* resulted from vicariance events that coincide with the Pleistocene climatic oscillations which fragmented humid montane forests in East Africa [45, 54, 55, 68, 69]. The savannas separating their current ranges were substantially stable even across glacial cycles in the late Pleistocene [69]. The occurrence of *L. aquilus* and *L. verhageni* is also consistent with the endemism of *Crocidura newmarki* on Mt. Meru [70] and *Myosorex zinki* on Mt Kilimanjaro [71]. The divergence and dispersal of the NONETHFLAVO1 ancestor coincided with temporary biogeographical contacts between the Ethiopian Highlands and other Afromontane forests in the early Pleistocene [69]. After initial colonization, montane forests were again fragmented by climatic oscillations, in the process facilitating allopatric speciation. Similarly, the patchy distribution of *Praomys delectorum* across the Eastern Arc Mountains and Southern Montane Forests was probably driven by comparable vicariance events [68]. The higher genetic diversity within *L. kilonzoi* (Table 1) suggests it remained in the ancestral range after diverging from the ancestor of *L. aquilus* + *L. verhageni*. Similar divergence and diversity patterns were observed for the forest-dependent *Praomys delectorum* [68], where the MRCA of populations from the Eastern Arc Mountains predated those from Mt. Kilimanjaro and Mt. Meru and correlated with genetic diversity.

#### The northern part of NONETHFLAVO:* L. stanleyi *and *L. zena*

The sister clades from the Kenyan and Ugandan highlands, *L. zena* and *L. stanleyi,* comprise the northern part of the NONETHFLAVO members. Our sample coverage of these two clades was the most comprehensive to date and substantially extend their known ranges. *Lophuromys zena* [31], thought to be endemic to the higher elevations of central Kenya [12, 14], occurs in all the stably humid ecosystems in Kenya, including Loita Hills forests in the southeast of their range to western (Mt. Elgon, Cherangani Hills, and Kakamega Forest) and southwestern (Victoria Basin – Yala Swamp) Kenya. The distribution of *L. zena* overlaps with *L. stanleyi* and *L. ansorgei* in the Kakamega Forest and with *L. ansorgei* in Yala Swamp. This distribution agrees with Onditi et al*.* [72] who noted that *L. zena* was more widespread in Kenya than previously known [12, 14]. The range of *L. stanleyi* is also much more extensive than previously described. Sabuni et al*.* [53] extended the range of *L. stanleyi* (delimited by mtDNA) into northwestern Tanzania beyond its Mt Rwenzori type locality [12], where it was thought to be restricted. Here we provide evidence that *L. stanleyi* occurs through much of Uganda, spanning southeastern South Sudan and northeastern Uganda forests eastwards to the Kakamega Forest in Kenya (its eastern limit) and south into northern Rwanda. The ‘Karamoja/Uganda gap’ [47, 73] was not a barrier to the dispersal of *L. stanleyi* through Uganda to connect the Kenya Highlands and Albertine Rift montane forests, as was the case for the forest-dependent *Hylomyscus* [47, 74]. Generally, the sister relationship of *L. zena* and *L. stanleyi* (minimal *CYTB* divergence) reinforces biogeographic affinities between the Albertine Rift montane forests and the Kenya Highlands [47–49]. Furthermore, the occurrence of *L. zena* and *L. stanleyi* in both lowland and highland forests suggest a phylogeographic pattern shaped also by an opportunistic ecological strategy, unlike true forest-specialists such as the *Hylomyscus denniae* and *Sylvisorex granti* groups that are restricted to high-elevation forests [47, 48]. The biogeographies of *L. zena* and *L. stanleyi* mirror patterns similar to the more widespread *Praomys jacksoni* which colonized both montane and lowland forests. However, the absence of a taxonomic structure based on *IRBP* reiterates the need to apply genomic analyses, especially in zones of secondary contacts, such as in Kakamega, to shed light on the level of reproductive isolation and taxonomic validity.

#### The southern part of NONETHFLAVO: *L. machangui* and *L. sabuni*

These two significantly supported sister clades correspond to *L. machangui* and *L. sabuni,* both described by Verheyen et al*.* [14] from Mount Rungwe and the Mbizi Mountains (Ufipa Plateau), respectively. Their sister relationship and late Pleistocene divergence coincide with the split of *L. verhageni* and *L. aquilus,* attributable to the late Pleistocene expansion of moist forests that likely enabled them to disperse to the current ranges, whose suitability was later restricted to highland forests. Overall, the distribution of *L. machangui* and *L. sabuni* reveals biogeographical trends that both coincide and contrast with other small mammals in the region, suggesting that other taxon-specific functional traits, such as dispersal ability and habitat specificity versus generality also influenced their evolutionary radiation. For instance, the distribution of *L. machangui* suggests the Makambako Gap has not barred its dispersal, similar for other small mammals including *Myosorex kihaulei* [75], but has barred the dispersal of *Praomys delectorum* [68] and *Otomys lacustris* [76]. Within the range of *L. sabuni*, Kerbis Peterhans et al*.* [73] recently described two species in the genus *Hylomyscus, Hylomyscus stanleyi* from Mbizi Forest Reserve and *Hylomyscus mpungamachagorum* from Mahale National Park, suggesting that the so-called Karema Gap was a barrier to the dispersal of these *Hylomyscus* species but not to the dispersal of *L. sabuni*. Overall, the close craniodental and genetic affinity between *L. sabuni* and *L. machangui* to each other in comparison with other members of the NONETHFLAVO2 subgroup suggests they have experienced somewhat similar ecological selection resulting in convergent ecomorphological characteristics [77]. The craniodental and genetic affinities between *L. sabuni* and *L. machangui* also concur with the floral and faunal affinity between the Southern highlands of the northern end of Lake Malawi and the Mbizi Forest, attributed mostly to the absence of a substantial biogeographical barrier between them. More studies are needed to delineate genetic differentiation across the range of *L. machangui* and *L. sabuni*, and detail how isolation by distance and geographical features have impacted their dispersal.

#### The western part of NONETHFLAVO: *L. dudui* and *L*. *rita*

The *L. dudui* clade comprised samples from the northeastern DRC montane highlands of the Albertine Rift –Rwenzori Mountains, westwards to the Kisangani – Bomane – Yaenero areas. This distribution leaves a ca. 480 km sample gap between the eastern limits (Epulu – Tshiabirimu – Ituri) and western limits near Bomane on the right bank and Boende on the left bank of the Congo River. The inclusion of samples from both sides of the Congo River in the *L. dudui* clade modifies the original description as well as consequent accounts of *L. dudui*, where it has been described to be restricted between the right bank of the Congo River and the western foothills of the Albertine Rift mountains [12, 14]. The current range of *L. dudui* resembles that of *Praomys mutoni* and *Praomys jacksoni* [55] both of which occupy lowland forests on both banks of the Congo River in the Kisangani region [55, 78]. Morphologically, *L. dudui* is easily diagnosable from the nearby NONETHFLAVO2 members due to its distinctly small skull (Fig. 6, Additional file 2: Table S1), consistent with previous findings [12, 14]. The *L. dudui* range overlaps with that of *L. rita*, which was assigned to samples spread over an expansive area in the Congo Basin, spanning southwestern DRC (Kinshasa) to the northeast (Kisangani, left bank of Congo River) and southwards to northwestern Zambia. Although we are unable to make skull comparisons with the holotype, this clade forms a well-defined mtDNA lineage, probably representing the *L. rita* described by Dollman [31] from south of Lake Tanganyika in NE Zambia (Mporokoso) and Lufupa River, Katanga, DRC. Despite its expansive range, *L. rita* appears bound to the central Congo basin by the Congo and Lualaba Rivers, which have likely limited its dispersal, like *Praomys minor* in the central Congo Basin [55]. Our geographic sampling of *L. rita* is notably sparse relative to its distribution and more surveys are necessary to resolve its full range and genetic diversity. Importantly, a formal taxonomic reassessment is required to validate the morphological relationship of the *L. rita* clade with the holotype and topotypes.

#### *Lophuromys makundii*

Specimens attributed to the monophyletic *L. makundii* derive from Mount Hanang (type locality) northwards over Lake Manyara and Ngorongoro crater to Mt Kitumbeine. Several ‘unsuitable’ dry corridors which currently isolate *L. makundii* from Eastern Arc Montane forests, Albertine Rift Mountains, Kenyan Highlands, and even the nearby Mount Kilimanjaro and Mount Meru seem to have impacted its dispersal after the initial colonization event. However, the occurrence of *Crocidura montis, Crocidura hildegardeae, Otomys angoniensis, Grammomys dolichurus/macmillani, Graphiurus murinus,* and *Praomys delectorum* in similar habitats as *L. makundii* in the north-central Tanzania region [53, 79] suggest that its biogeographical affiliation to other Eastern Afromontane forests in the region is recent. The relatively isolated range of *L. makundii* likely imposed a more rigid barrier to genetic exchange with other lineages after divergence [80] and might explain why it is the only other clade in the NONETHFLAVO, besides *L. kilonzoi,* that retains monophyly in the *IRBP* tree (Additional file 1: Fig. S4). Still, the short divergence time from and possible sister relationship to either *L. dudui* or *L. laticeps* show that it is more closely affiliated to the Albertine Rift clades than the NONETHFLAVO1 members. As such, *L. makundii* probably colonized its current range when moist forests connected the currently isolated volcanic mountains during the late Pleistocene climate fluctuations.

#### *Lophuromys* cf. *cinereus *and *L*. *laticeps*

The *L.* cf. *cinereus* samples overlap the Kahuzi-Biega National Park locality from where Dieterlen and Gelmroth [28] described *L. cinereus.* Following the initial proposal by Dieterlen [11] that the external and craniodental distinctness used by Dieterlen and Gelmroth [28] to describe *L. cinereus* were, in fact, morphotypes of *L. laticeps,* there has since been no formal taxonomic reassessment of its validity [6, 12]. Our mtDNA*,* nuclear (*IRBP*), and craniodental tests showed similar differences between the *L.* cf. *cinereus* and *L. laticeps* clades comparable to the distances within and between other NONETHFLAVO2 clades (Table 1), including the sister clade, *L. rita*. The *L.* cf. *cinereus* skulls overlapped most with *L. laticeps, L. dudui,* and *L. stanleyi,* consistent with the earlier rationale for its synonymy [6, 12]. A formal taxonomic revision of *L.* cf. *cinereus,* is needed to validate and update its distribution, and genetic and phenetic relationship to other NONETHFLAVO members. Such a revision would update the occurrence of *L. cinereus* (herein as *L. cf. cinereus*), which was perceived restricted to the type locality [28, 81], to extend from Kahuzi-Biega National Park to the Itombwe Massif and southwards ca. 300 km to Mt. Kabobo—on the western shore of Lake Tanganyika. Thomas and Wroughton [29] considered *L. laticeps* as a morphologically unique lineage among its close relatives allied to *L. aquilus* [23] due to a broader lower braincase and shorter palatal foramina. Our *L*. *laticeps* and *L.* cf. *cinereus* skulls had the broadest BBC, while *L. laticeps* had one of the shortest PPL in the NONETHFLAVO dataset (Additional file 2: Table S1). The *L. laticeps* clade is also genetically well-differentiated, comparably, to close relatives—*L.* cf. *cinereus, L. stanleyi,* and *L. laticeps* (Table 1). There is a need to formally reassess the taxonomy of *L.* cf. *cinereus* and *L. laticeps,* to clarify and update their distinctness from other lineages in the NONETHFLAVO.

#### *Lophuromys margarettae, L. rubecula, *and *L. major*

No genetic OTUs could be matched to *L. margarettae, L. rubecula,* or *L. major*, despite sampling from their respective ranges—Mathews Range, Mount Elgon, and proximity of Ubangi River. *Lophuromys margarettae* was described by Heller [82] from the Mount Gargues (Mathews range), north-central Kenya, with Verheyen et al*.* [12], Verheyen et al*.* [14] asserting its presence on the lower elevations of the Kenya highlands. However, Onditi et al*.* [72] did not record *L. margarettae* in the entire elevation gradient of Mount Kenya (ca. 1700–4000 m). In the current study, the samples from Kaptagat that Verheyen et al*.* [14] assigned to *L. margarettae* are completely nested within the *L. zena* clade, including those from the nearby Mau Forest fragments. During this study, despite ~ 500 trap nights (standard trapping protocol using Sherman live traps) at intermediate elevations of the Mathews Range (1,210–1,930 m), not a single *Lophuromys* was captured. Although we cannot challenge the taxonomic validity of *L. margarettae* in the Mathews Range yet, it is absent from all the localities where *L. zena* was sampled—virtually all the wet highlands of Kenya. It may be that ongoing forest degradation and changing climates have led *L. margarettae* to shift range and thus become more rare. More surveys of the higher, more intact forest of the Mathews Range are required to resolve with certainty whether *L. margarettae* is still resident in the area or is simply an *L. zena* variant.

Similarly, *L. rubecula* described by Dollman [27] is another species we were unable to confirm without new material. Our Mt. Elgon samples cluster genetically and craniodentally with *L. zena.* However, we lacked samples from other parts of the Mt Elgon ecosystem, without which we cannot dismiss *L. rubecula*’s occurrence or its validity. Future surveys of Mt Elgon should employ elevational stratified sampling transects on the Kenya and Uganda sides to substantiate the occurrence limits (or the absence thereof) of *L. rubecula*.

Finally, we were also unable to verify the validity of *L. major,* which was described by Thomas and Wroughton [29] from the Bwanda area, Ubangi River, DRC. The ranges of the presupposed nearest congeners—*L. dudui* and *L. rita* are considerably south of its type locality and without new material from the area, we cannot verify the validity of *L. major* or approximate its relationship to other species in the *Lophuromys* genus.

### Implications of discordances between genes’ trees and the species tree

Despite providing a comprehensive scenario of the systematics, taxonomy, and historical biogeography of the NONETHFLAVO members, our discussions should be interpreted with caution; within the limitations of being driven mainly by *CYTB*, the genes’ trees versus species tree discordances, and the posterior and bootstrap supports. When compared with existing taxonomic accounts of *Lophuromys* species [12–17, 72], the *CYTB* tree provided an informative, resolved, and reliable topology of species limits, which was, however, discordant with the *COI* and *IRBP* gene trees and species tree of the concatenated alignment [*CYTB* + *COI* + *IRBP*]—Additional file 1: Fig. S6. The resolved clades following species delimitation (Fig. 1), for instance, generally correspond to the mtDNA trees’ topologies of Lavrenchenko et al*.* [17], Verheyen et al*.* [14], and Komarova et al*.* [16], as well as the morphometric and biogeographic accounts in *Lophuromys* taxonomic reassessments [12–15] and recent mammal checklists [4–7]. The discordances in tree topologies fit several theoretical discussions concerning phylogenetic reconstructions from multi-locus alignments, and which could be specific to the genus *Lophuromys* and the genes used. For one, *COI* and *IRBP* had very low phylogenetic informativeness—20% and 8% parsimony informative sites, respectively, compared to *CYTB* (40% parsimony informative sites); which probably impacted their phylogenetic relevance and that of the gene tree. Still, factors such as incomplete lineage sorting of *IRBP*, incomplete taxon sampling, and incomplete gene sampling could also have caused the phylogenetic informativeness/lack of resolution of the *COI* and *IRBP* and species trees. Nabhan and Sarkar [83] discussed the challenges of a tradeoff between taxon versus character (gene) sampling towards improving phylogenetic resolution, particularly because phylogenetic errors are negatively correlated with both taxon sample coverage and character sample coverage [84]. Hillis et al*.* [84], for instance, disagreed with adding more characters compared to adding more taxa, while Lambert et al*.* [85] noted that increasing taxon sample coverage may be more important than increasing character sample coverage when estimating species trees from concatenated sequence alignments. Notably, our reliance on the *CYTB* alignment over the concatenated genes’ alignment concurs with studies such as Gabriel et al*.* [86] where more precise phylogenies were obtained using fewer genes, and Tsang et al*.* [87] where the multi-locus phylogeny was less resolved and not representative of true phylogenetic associations. The ultimate relevance of taxon and gene sampling inherently depends on the context of where and how phylogenetic inference is applied [84] since including incomplete genes and/or genes with missing data (due to the shorter sequences and samples that were not successfully sequenced for all three genes) might increase phylogenetic resolution and branch/node support compared to excluding them [88].

## Conclusion

Despite being one of the most widely occurring and abundant rodents in east-central and east African montane and lowland rain forest habitats, the taxonomy and historical biogeography of the NONETHFLAVO members remain poorly understood. Our utilization of the *CYTB* gene to reconstruct the phylogenetic relationships of the genus *Lophuromys* and combined mitochondrial and nuclear genes and morphometrics (geometric and linear characters) to analyze the systematics of the NONETHFLAVO substantially extends the understanding of their taxonomy and evolutionary radiation. While most of the species recognized previously based on morphology are supported as well geographically structured mtDNA lineages, they lack stable informative craniodental characters capable of reliably assigning samples to putative species units a priori. The NONETHFLAVO colonized its current range over two independent dispersal events out of Ethiopia in the early Pleistocene, with the two resulting subgroups remaining respectively monophyletic but nested in the ETHFLAVO members. While our study has provided a comprehensive scenario for the evolution, phylogeography, and genetic differentiation of the NONETHFLAVO, a formal taxonomic harmonization based on more comprehensive genomic characterization of the genus is required to ascertain the full extent and influence of mitochondrial-nuclear phylogenetic incompatibilities, as accomplished recently for the ETHFLAVO members [16]. Ultimately, such a comprehensive genomic phylogenetic approach, even in the absence of craniodental data, is likely to reliably delimit the unique population pools corresponding to valid species and the resolution of species groups. Currently, the ranges of the NONETHFLAVO members are restricted to ecosystems with stable annual precipitation regimes, which are susceptible to habitat degradation and global climate changes. The increasingly fluctuating climatic regimes, warming climates, and continued habitat fragmentation are likely to degrade habitat conditions for most clades, fragmenting further their distributions, and resulting in substantial range shifts and or loss of habitat. This would reciprocally drive divergent eco-evolutionary trait and genetic adaptative responses between sympatric and parapatric close relatives, with taxonomic implications that are essential from a biodiversity conservation point of view.

## Methods

### Sampling

We compiled three datasets for the combined genetic and morphometric analyses. Sampling across the genus *Lophuromys* was possible for *CYTB* only and covered the currently known range of the genus (Fig. 2). Sampling for the full dataset (*CYTB,* *COI, IRBP,* and linear and geometric data) was possible for the NONETHFLAVO members only, for which we sampled the known range, representing type localities (or their environs) of all species currently classified under or associated with the group (Fig. 2, Additional file 1: Fig. S2, Additional file 3: Table S4). The skulls are deposited at the Field Museum of Natural History, Chicago, USA (FMNH), Kunming Institute of Zoology, Kunming, China (KIZ), and National Museums of Kenya, Nairobi, Kenya (NMK).

### Genetic data

Total DNA was extracted from muscle or liver tissue preserved in absolute ethanol at -80 °C using the sodium dodecyl sulfate method [89]. The DNA was PCR-amplified using gene-specific primer pairs (Additional file 2: Table S3). The PCR reaction template comprised of 20 µl volumes (0.5 µl primer pairs, 10 µl PCR Master Mix, 8.5 µl water, and 0.5 µl DNA template); the cycling temperature, time settings, and primers were specified as shown in Additional file 2: Table S3. The amplified product was sequenced in forward and reverse directions using the ABI Genetic Analyzer (Applied Biosystems), assembled in Geneious Prime® 2020.2.4 (https://www.geneious.com, Accessed September 2020), and aligned in Aliview v.1.26 [90] using MUSCLE [91]. After dropping duplicates and sequences with a high ratio of gaps/ambiguous bases, we retained 803 *CYTB* sequences, of which 316 were newly generated, and the rest downloaded from GenBank [92] and the African Mammalia database [93] (Additional file 3: Table S4). From the new *CYTB* sequences, we subsampled from the unique haplotypes and extracted 138 *COI* and 100 *IRBP* sequences, which were aligned separately and concatenated in SequenceMatrix [94]. The alignment of concatenated loci was available for the NONETHFLAVO members only and comprised 91 sequences, 3088 bp long (1140 bp *CYTB*, 717 bp *COI*, and 1231 bp *IRBP*), after matching similar sample identifications. We confirmed that there were no premature stop codons, indels, or heterozygous bases in MEGA X v.10.1.8 [95] and resolved heterozygous bases in the *IRBP* alignment using PHASE [96] in DnaSP v.6 [97]. The sequences used in the molecular analysis are included in Additional file 3: Table S4, of which the unique new sequences were submitted to GenBank (https://www.ncbi.nlm.nih.gov/genbank/), accession numbers MW464441 - MW464606.

### Morphometric data

Morphometric variation among the NONETHFLAVO members was inferred using a linear dataset of 725 skulls [310 ♀, 363 ♂, and 23 unsexed specimens] and a geometric dataset of 635 two-dimensional cranial images [278 ♀, 338 ♂, and 19 unsexed specimens] (Additional file 3: Table S4). The samples were age-classified based on the stage and pattern of M^3^ wear into three age classes: *young adults*—fully erupted M^3^ but very little to no visible wear, *adults*—medium wear on M^3^, and *old adults*—medium to extensive M^3^ wear. Consequently, the geometric dataset comprised 29% young adults, 40% adults, and 31% old adults, while the linear dataset comprised 28% young adults, 41% adults, and 31% old adults. The samples’ assignment to age and sex categories was used to explore and control the effects of skull size variation (ontogeny and sexual dimorphism), which can obscure sought differences between taxonomic groups [98, 99]. We used TPSUtil v.1.74 and TPSDig2 v.2.30 [100] to digitize 37 landmarks on the 2-dimensional skull images (Additional file 1: Fig. S8) and processed the resulting dataset in MorphoJ v.1.07a using Generalized Procrustes Analysis (GPA). The GPA untangles shape and size to produce centroid size (CS) and Procrustes coordinates. For the linear craniodental variation analysis, we used the same measurements and extraction techniques as in Onditi et al*.* [72].

### Data analysis

#### Phylogenetic analysis

The mitochondrial phylogeny of the genus *Lophuromys* was reconstructed from an alignment of 241 *CYTB* sequences (1140 base pairs long, 711 distinct patterns, 443 parsimony-informative, 88 singleton sites, and 609 invariant sites), which included single longest sequences of each haplotype identified in the initial 803 sequences. The alignment represented all the species currently recognized in the genus *Lophuromys* [5, 22], except *L. medicaudatus,* *L. eisentrauti,* and *L. dieterleni* for which we could not obtain representative new material or publicly available sequences. Sequences of *Acomys ignitus, Deomys ferrugineus,* and *Uranomys ruddi*, downloaded from GenBank, were used as outgroups. We used maximum likelihood (ML) and Bayesian inference (BI) methods for phylogenetic reconstructions, based on a GTR + F + G4 model of nucleotide substitution, which was identified as the best-fitting under the Bayesian information criterion (BIC) in ModelFinder [101]. The ML analysis was performed using IQ-TREE v.1.6.12 [102] in PhyloSuite v.1.2.2 [103] using 100,000 ultrafast bootstrap replicates [104] to estimate branch support (BS). The BI analysis was performed in MrBayes v.3.2.7a [105] with two independent runs involving 10 million generations each, sampled every 1000th run, using the reversible-jump Markov chain Monte Carlo (MCMC) [106] to estimate posterior probability support [PP]. The BI results were visualized in Tracer v.1.7.1 [107] to diagnose convergence using the effective sample size values (ESS), with values > 200 considered adequate. The majority-rule consensus tree was annotated after discarding 25% as burn-in. The resulting trees from the ML and BI analyses were graphically edited in FigTree v.1.4.4 [108].

#### Species delimitation and genetic diversity analysis

Initial principal component analysis (PCA) tests on the linear dataset showed craniodental characters did not cluster samples consistent with current taxonomic accounts in the literature (Additional file 1: Fig. S9). Therefore, we used the *CYTB* dataset to define operational taxonomic units (OTUs), representing biological units to delimit species limits (clades). We used delimitation methods that can reliably identify common species units without prior assignment of samples to taxonomic units and implemented both tree-based and distance-based algorithms. For tree-based species delimitation, we used the branch-cutting method (BCUT, Mikula [109]), the multi-rate Poisson Tree Processes algorithm (mPTP, Kapli et al*.* [110]), and the single threshold general mixed Yule coalescent model (GMYC, Fujisawa and Barraclough [111]). We used the genus-wide ML tree as input in BCUT and mPTP analyses and a time-calibrated tree reconstructed in BEAST2 v.2.6.3 [112] for GMYC. The BCUT and GMYC analyses were performed in R v.4.0.3 [113] using functions provided by the author for the former and the splits package [114] for the latter. The mPTP analysis was implemented using the command-line options with four MCMC runs of 500 million generations, each sampled every 50,000 runs with a 10% burn-in, with convergence confirmed from a visual inspection of the combined likelihood plot. Finally, the distance-based delimitation was performed using the Automated Barcode Gap Discovery method (ABGD, Puillandre et al*.* [115]). The same genus-wide *CYTB* alignment for the phylogenetic reconstructions was used as input. The analysis was run in the ABGD web server (https://bioinfo.mnhn.fr/abi/public/abgd/abgdweb.html, Accessed 10 November 2020) using the K80 Kimura measure of distance, 0.001–0.1 prior bounds for intraspecific divergence, and a 0.75 relative gap width. Species names of the resolved clades—Fig. 1 and Additional file 1: Fig. S1—were extracted from previous classifications in literature by matching with recently clarified *Lophuromys* taxonomies [12–17] and the mammal checklists [4–7, 32, 33].

We used haplotype networks to inspect further the genealogical relationships between the delimited OTUs. Haplotype networks visualize genetic relationships among haplotypes, and because they do not force branching schemes, they may reflect evolutionary relationships better than the phylogenetic trees [116]. The haplotype networks were reconstructed using haplotypes generated in DnaSP and visualized in PopART v.1.7 [117] based on the Median Joining Network algorithm [118]. The genetic divergence within and between the delimited OTUs was explored using various indices of genetic diversity estimated in DnaSP, including the number of haplotypes, haplotype diversity, and nucleotide diversity. The genetic distances between and within the resolved OTUs/clades were estimated in MEGAX based on the number of nucleotide differences per site averaged between sequence pairs (uncorrected *p*-distances).

#### Estimation of divergence times

The divergence between main clades in the genus *Lophuromys* was inferred using the genus-wide *CYTB* alignment based on the coalescent-based approach in BEAST2. We applied secondary calibrations of the most recent common ancestor (MRCA) since *Lophuromys* has no fossil record. Two secondary calibration points were specified; the divergence between the *L. sikapusi* and *L. flavopunctatus* groups, which was estimated by Aghova et al*.* [119] to ca. 3.71 million years ago [Mya] (confidence: 2.66–5.05) and the root node of the subfamily *Deomyinae* (having included sequences of *Acomys ignitus, Deomys ferrugineus, Uranomys ruddi* as outgroups). According to Aghova et al*.* [119], diversification within *Deomyinae* commenced ca. 13.8 Mya (95% highest posterior density interval [HPDI]: 12.04–16.01). We used lognormal priors with a mean of 1.31 and standard deviation (SD) of 0.1 (median 3.71 Mya) for the divergence between the *L. sikapusi* group and *L. flavopunctatus* group and a mean of 2.628 and SD of 0.06 (median 13.8 Mya) for the MRCA of the *Deomyinae* subfamily members. Because the three genes, *CYTB*, *COI*, and *IRBP* were available for the NONETHFLAVO members only, we estimated a species tree of the group using the StarBEAST2 package [120] of BEAST2. The time-calibration was based on the divergence between the *L. sikapusi* and *L. flavopunctatus* groups as specified above, following the inclusion of an *L. sikapusi* sequence as an outgroup. Two separate and unlinked substitution, clock and tree models corresponding to the mitochondrial (*CYTB* + *COI*) and nuclear (*IRBP*) loci were set, fitted with uncorrelated lognormal clock and Yule speciation models. The time-calibrated phylogeny of the genus *Lophuromys* and the NONETHFLAVO species tree were implemented with two MCMC runs, each 100 million generations-long, sampled every ten thousand runs. The sampling convergence was assessed in Tracer; all the parameters had ESS values > 400. The runs, including tree and log files, were combined in LogCombiner after discarding 10% as burn-in. The trees were summarized in TreeAnnotator and graphically edited in FigTree.

#### Biogeographical analysis

We reconstructed species ancestral ranges in RASP v.4.2 [121, 122], based on the dispersal-extinction cladogenesis (DEC) model [123] which was selected with the BioGeoBEARS R package [124] as best-fitting to our dataset. The DEC model uses a species tree (with branch lengths scaled to evolutionary divergence times) and the geographical areas where the species (tree tips) occur to estimate ancestral ranges. The input tree was reconstructed from a reduced (single sequences from each GMYC-delimited OTU) time-calibrated genus-wide *CYTB* tree based on the same secondary calibrations as above. The major biogeographic ecoregions were defined according to Dinerstein et al*.* [125] [https://ecoregions2017.appspot.com/—Accessed 5th November 2020], with slight modifications. A total of six ecoregions were used; Albertine Rift montane forests, Guinea-Congo forests, East African montane forests, Eastern Arc forests, Ethiopian montane forests, and Southern Rift Montane forests. Because neither the ETHFLAVO nor NONETHFLAVO are monophyletic and range overlap exists between the *Kivumys* group*, L. sikapusi* group, and NONETHFLAVO, dispersal was allowed between all the ecoregions.

#### Morphometric analyses

The linear variables were initially transformed by natural logarithms to enhance their multivariate normality. The presence of outliers was explored using Tukey's 1.5*IQR rule with a custom R script (http://goo.gl/UUyEzD, Accessed 1st October 2020). From the combined 725 skulls for linear morphometry, < 2% outliers existed for any of the 14 measurements across species groups, therefore, they were simply replaced with the respective group mean for each measurement. In the geometric dataset, outliers were checked for in MorphoJ for each species group; only a single sample was identified as an outlier, and it was simply excluded from consequent analyses. We controlled for the potential effects of allometry using residual analyses. In the geometric dataset, we regressed the shape variables (Procrustes coordinates) on centroid size (CS) in MorphoJ, and the resulting residuals were used as the new shape variables for consequent discriminant and multivariate analyses. In the linear dataset, we performed an initial PCA of the 14 linear measurements to extract the first axis (PC 1) which accounted for 71% variance. The linear measurements were then regressed on PC 1, with the residuals used as linear variables for consequent analyses.

The craniodental differences between clades were estimated using discriminant function analysis (DA) in IBM SPSS Statistics v25 based on the within-group covariance matrices for both linear and geometric datasets. In the DA, each group was assumed to have equal prior probabilities, so that cases were equally assignable to any group regardless of sample size. To test how classification accuracy compared to random assignment, we used the leave-one-out cross-validation model, where a discriminant function classifies cases based on all other cases except itself. Discriminant analysis is preferable when delimitating interspecific morphological differences due to its ability to estimate the combination of characters that best distinguish groups [99, 126]. Statistical significances of between-clade differences were estimated using permutational multivariate analysis of variance (PerMANOVA), with pairwise PerMANOVAs—between clade pairs—used as post hoc tests. The significances of comparisons were computed by permutation of group membership (9999 replicates) and determined based on *F* values and Bonferroni-corrected *p* values. We also used dendrograms of group mean clusters following MANOVA (performed using the manovacluster MATLAB function [www.mathworks.com/help/stats/manovacluster.html?s_tid=srchtitle, Accessed 1st October 2020] based on the single linkage method) to visualize the multivariate craniodental relationships between clades.

## Supplementary Information

**Additional file 1: Figure S1.** The phylogeny of the genus *Lophuromys* inferred from the *Cytochrome b* gene using Bayesian Inference in MrBayes. There were a total of 238 sequences in the analysis, representing all unique haplotypes from the initial alignment of 803 sequences. The numbers above branches represent the percentage posterior probability values. **Figure S2.** Map showing the type localities (red, green outlined crosses '+') of the non-Ethiopian *L. flavopunctatus* members, with the corresponding species names labeled in red fonts. The sampling points of samples used in the study are also shown, outlined to illustrate their distribution extents. **Figure S3.** A Maximum likelihood phylogeny of the non-Ethiopian *L. flavopunctatus* members inferred from the concatenated mitochondrial genes (*Cytochrome b gene* + *cytochrome oxidase subunit 1*) in IQ-TREE. The taxa labels represent the consensus species identities of main OTUs identified by species delimitation. Values above branches represent percentage Ultrafast Bootstrap support values. **Figure S4.** A maximum likelihood phylogeny of species in the *L. flavopunctatus* group inferred from the *Interphotoreceptor retinol binding protein* gene in IQ-TREE. Taxa labels indicate the samples ID _ Locality _ species names. Similar label font colors represent the same clades. All taxa with 'FMNH' and 'KE' in their IDs were sequenced from the current study, the rest were downloaded from GenBank. Values above branches are the ultrafast bootstrap support percentages. **Figure S5.** Haplotype network structure in selected non-Ethiopian *L. flavopunctatus* members inferred from *Cytochrome b* using the Median Joining Network algorithm in PopART. The networks show genealogical relationships between sampling locality in the *L. zena, L. machangui,* and *L. zena* clades which were selected for being sampled from broader areas and more localities. The number of base substitutions between haplotypes is shown as branch hatch marks. The node sizes correspond to the haplotype frequency (number of samples per haplotype) and branch lengths are relative to the number of mutations between haplotypes. **Figure S6.** Time calibrated maximum clade credibility tree of evolutionary relationships and divergence times in the genus *Lophuromys* reconstructed from *Cytochrome b, Cytochrome oxidase subunit 1,* and *Interphotoreceptor retinoid binding protein* using secondary most recent common ancestor calibrations. Branch labels show the posterior probability support values, node labels illustrate the median divergence height (age), and node bars show the highest posterior density interval. **Figure S7.** Classification results following discriminant analysis in using linear and geometric craniodental datasets of the non-Ethiopian *L. flavopunctatus* members. Values indicate cross-validated (leave-one-our bootstrapping) percentage success by which samples were classified into a priori and predicted species groups. The shaded diagonal values indicate the success by which samples were predicted into their groups which correspond to distinct *Cytochrome b* clades. N = number of samples. See Fig. 6 in the main manuscript for clade-stratified classification results. **Figure S8.** Cranial landmarks used in geometric morphometric analyses of the non-Ethiopian *L. flavopunctatus* members. **Figure S9.** Principal component analysis (PCA) of linear measurements used in the study. The plots show how samples cluster based on the first and second component scores. The top left scatter plot shows samples do not cluster in a distinct pattern consistent with the taxonomic units currently acknowledged in literature (top right). The bottom left plot shows the variances accounted for by all the component loadings.

**Additional file 2: Table S1.** Summary statistics of linear cranial measurements and centroid size (CS) of species the non-Ethiopian *L. flavopunctatus* members. The values represent the 95% confidence interval mean | standard deviation | minimum–maximum values. **Table S2.** Summary of multivariate pairwise differences between the non-Ethiopian *L. flavopunctatus* members based on linear measurements and geometric craniodental landmarks inferred using permutational multivariate analysis of variance. Values represent pairwise PerMANOVAs between clade pairs as post hoc tests with the upper matrix showing the *F* values and the lower matrix showing the Bonferroni-corrected p values (statistically significant values are in bold). The test of PerMANOVA showed overall significant skull differences between clades in both the linear dataset (Total sum of squares: 35510; Within-group sum of squares: 29300; *F*: 15.13, *p*: 0.0001) and geometric dataset (Total sum of squares:0.3591; Within-group sum of squares: 0.3091; *F*: 10.1; *p*: 0.0001). **Table S3.** List of genes amplified showing the respective primers used and PCR reaction settings. F = forward primer, R = reverse primer. The thermal profile for *IRBP* amplification consisted of a touch-down annealing protocol from 52 to 56 °C.

**Additional file 3: Table S4.** A detailed list of all samples used in the study, with sampling coordinates, locality names, and external measurements, among other details.

## Data Availability

All data generated or analyzed during this study are included in this article and its supplementary information files. The sequences used in the molecular analysis are included in Additional file 3: Table S4, of which the unique new sequences were submitted to GenBank (https://www.ncbi.nlm.nih.gov/genbank/), accession numbers MW464441 - MW464606.

## Abbreviations

ABGD: Automated Barcode Gap Discovery method
BCUT: Branch-cutting species delimitation method
BI: Bayesian inference
BIC: Bayesian information criterion
BS: Bootstrap support
*COI*: Cytochrome c oxidase I
CS: Centroid size
*CYTB*: Cytochrome b
DA: Discriminant function analysis
ESS: Effective sample size values
FMNH: Field Museum of Natural History, Chicago, USA
GPA: Generalized Procrustes Analysis
GYMC: Single threshold general mixed Yule coalescent model
HPDI: Highest posterior density interval
*IRBP*: Interphotoreceptor retinol-binding protein
KIZ: Kunming Institute of Zoology, Kunming, China
M^3^: Third molar tooth
MANOVA: Multivariate analysis of variance
MCMC: Markov chain Monte Carlo
ML: Maximum likelihood
mPTP: Multi-rate Poisson Tree Processes algorithm
MRCA: Most recent common ancestor
Mt(s): Mountain(s)
mtDNA: Mitochondrial Deoxyribonucleic Acid
Mya: Million years ago
NMK: National Museums of Kenya, Nairobi, Kenya
OTU: Operational taxonomic unit
PerMANOVA: Permutational multivariate analysis of variance
PCR: Polymerase chain reaction
PP: Posterior probability support
