# Supplementary material for: Historical biogeography, systematics, and integrative taxonomy of the non-Ethiopian speckled pelage brush-furred rats (Lophuromys flavopunctatus group)
Source: BMC Ecol Evol. 2021 May 19;21:89. doi: 10.1186/s12862-021-01813-w (PMC8132446; doi:10.1186/s12862-021-01813-w)
Supplement: Supplementary file 1 — Additional file 1: Figure S1. The phylogeny of the genus Lophuromys inferred from the Cytochrome b gene using Bayesian Inference in MrBayes. There were a total of 238 sequences in the analysis, representing all unique haplotypes from the initial alignment of 803 sequences. The numbers above branches represent the percentage posterior probability values. Figure S2. Map showing the type localities (red, green outlined crosses '+') of the non-Ethiopian L. flavopunctatus members, with the corresponding species names labeled in red fonts. The sampling points of samples used in the study are also shown, outlined to illustrate their distribution extents. Figure S3. A Maximum likelihood phylogeny of the non-Ethiopian L. flavopunctatus members inferred from the concatenated mitochondrial genes (Cytochrome b gene + cytochrome oxidase subunit 1) in IQ-TREE. The taxa labels represent the consensus species identities of main OTUs identified by species delimitation. Values above branches represent percentage Ultrafast Bootstrap support values. Figure S4. A maximum likelihood phylogeny of species in the L. flavopunctatus group inferred from the Interphotoreceptor retinol binding protein gene in IQ-TREE. Taxa labels indicate the samples ID _ Locality _ species names. Similar label font colors represent the same clades. All taxa with 'FMNH' and 'KE' in their IDs were sequenced from the current study, the rest were downloaded from GenBank. Values above branches are the ultrafast bootstrap support percentages. Figure S5. Haplotype network structure in selected non-Ethiopian L. flavopunctatus members inferred from Cytochrome b using the Median Joining Network algorithm in PopART. The networks show genealogical relationships between sampling locality in the L. zena, L. machangui, and L. zena clades which were selected for being sampled from broader areas and more localities. The number of base substitutions between haplotypes is shown as branch hatch marks. The node sizes correspond to [file 12862_2021_1813_MOESM1_ESM.pdf]

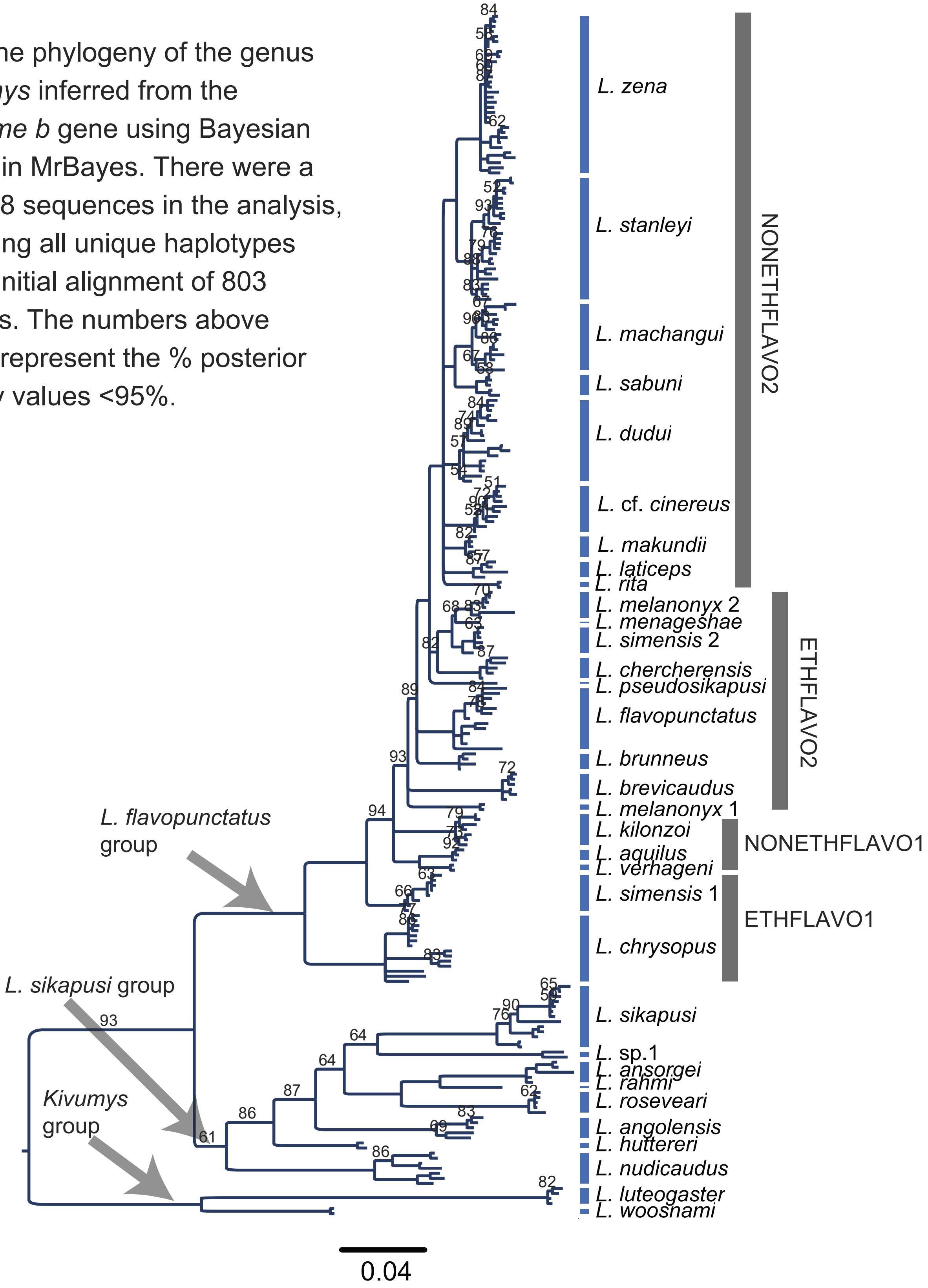

Fig. S1 The phylogeny of the genus *Lophuromys* inferred from the *Cytochrome b* gene using Bayesian Inference in MrBayes. There were a total of 238 sequences in the analysis, representing all unique haplotypes from the initial alignment of 803 sequences. The numbers above branches represent the % posterior probability values <95%.

Fig. S2 Map showing the type localities (red, green outlied crosses '+') of species described under the non-Ethiopian *flavopunctatus* group, with the corresponding species names labeled in red fonts. The sampling points of samples used in the study are also shown, outlined to illustrate their distribution extents.

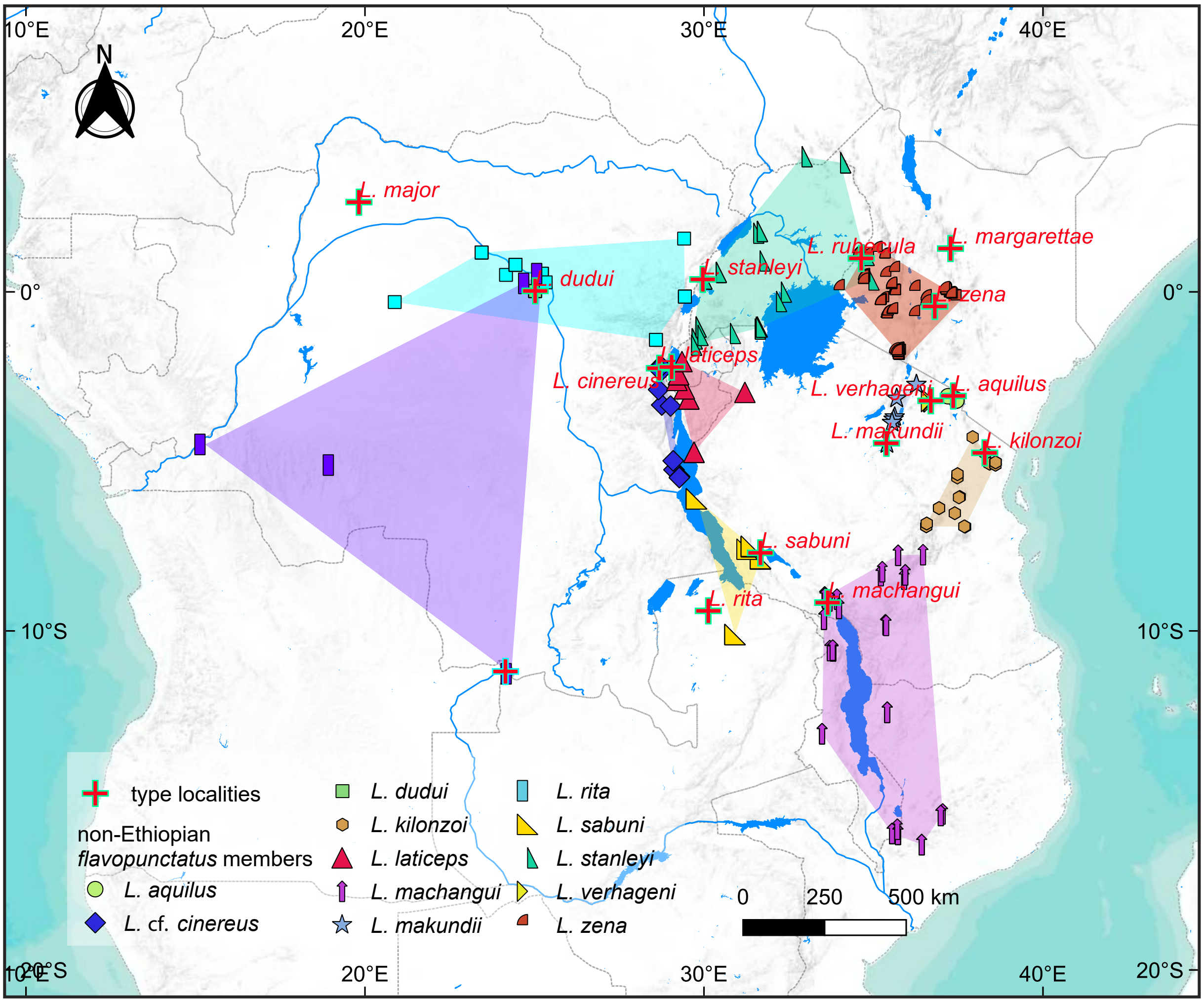

Fig. S3 A Maximum likelihood phylogeny of the non-Ethiopian *L. flavopunctatus* members inferred from the concatenated mitochondrial genes (Cytochrome b gene + cytochrome oxidase subunit 1) in IQ-TREE. The taxa labels represent the consensus species identities of main OTUs identified by species delimitation. Values above branches represent percentage Ultrafast Bootstrap support values.

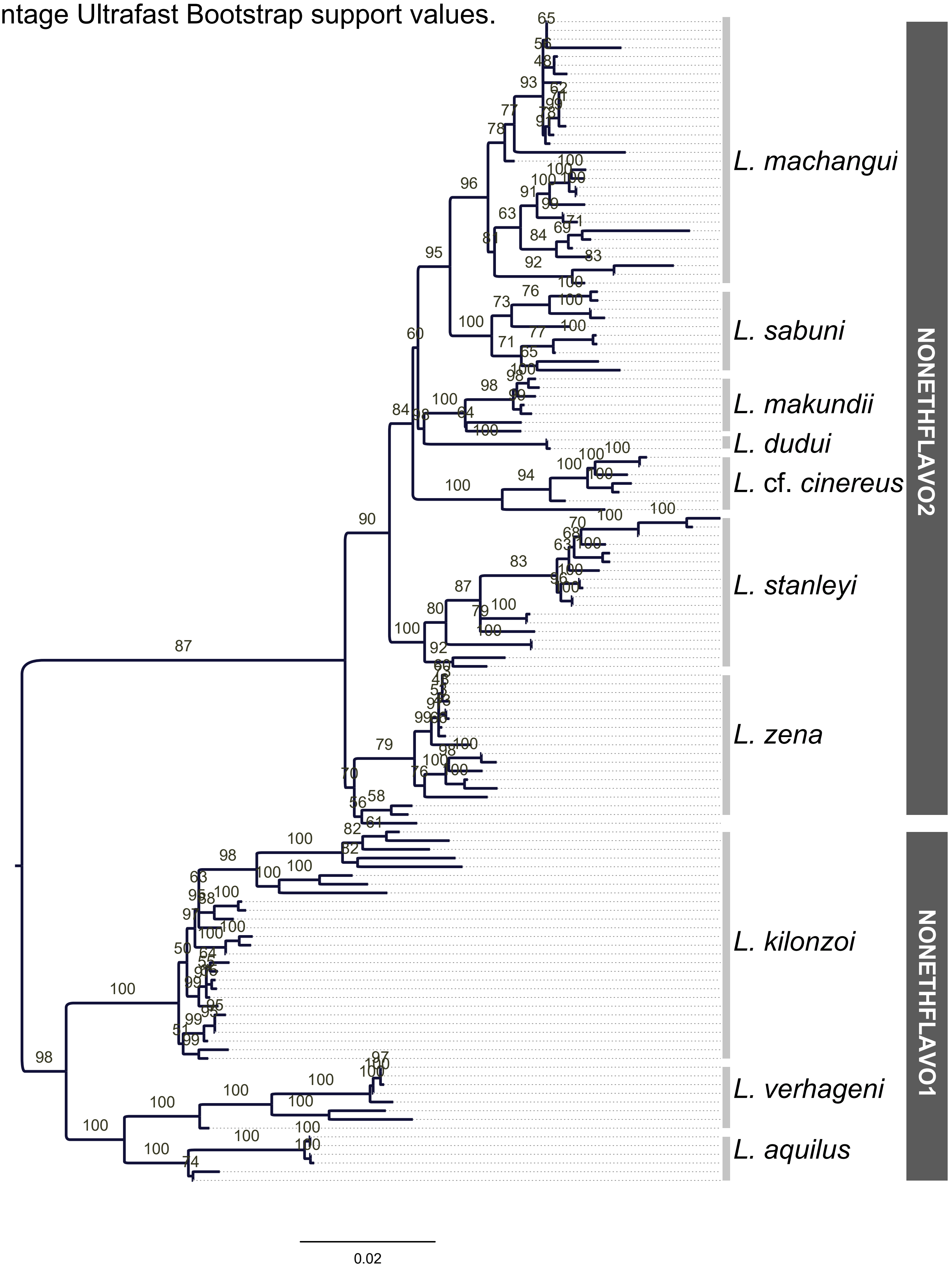

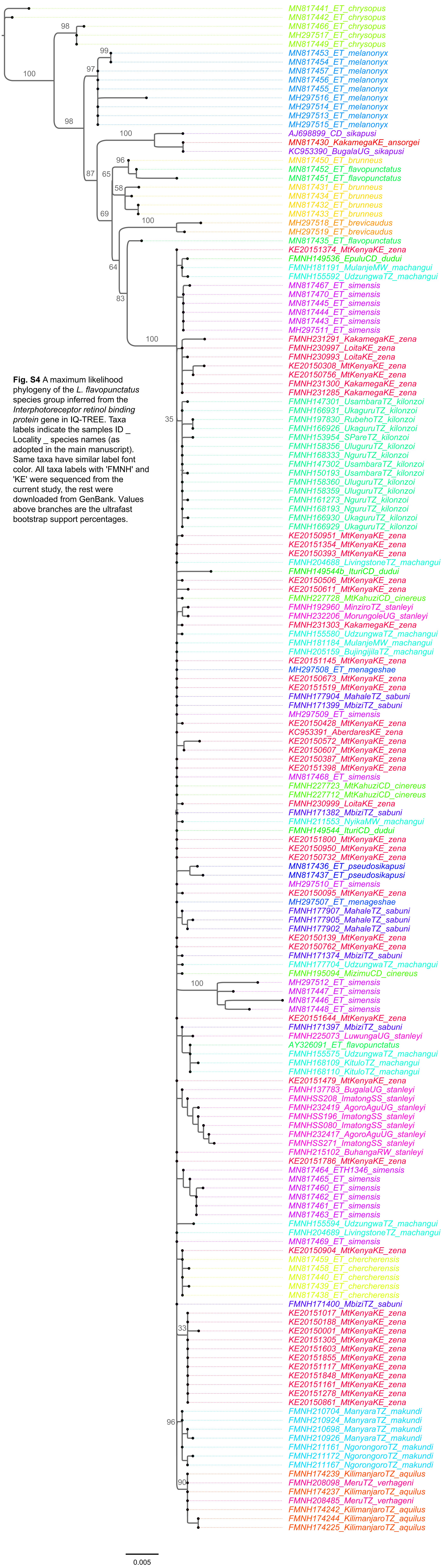

Fig. S4 A maximum likelihood phylogeny of the *L. flavopunctatus* species group inferred from the *Interphotoreceptor retinol binding protein* gene in IQ-TREE. Taxa labels indicate the samples ID _ Locality _ species names (as adopted in the main manuscript). Same taxa have similar label font color. All taxa labels with 'FMNH' and 'KE' were sequenced from the current study, the rest were downloaded from GenBank. Values above branches are the ultrafast bootstrap support percentages.

Fig. S5 Haplotype network structure in selected non-Ethiopian *L. flavopunctatus* members inferred from *Cytochrome b* using the Median Joining Network algorithm in PopART. The networks show genealogical relationships between sampling locality in the *L. zena, L. machangui,* and *L.* stanleyi clades which were selected for being sampled from more localities. The number of base substitutions between haplotypes are shown as hatchmarks on branches. The node sizes correspond to the haplotype frequency (number of samples per haplotypes) and branch lengths are relative to the number of mutations between haplotypes.

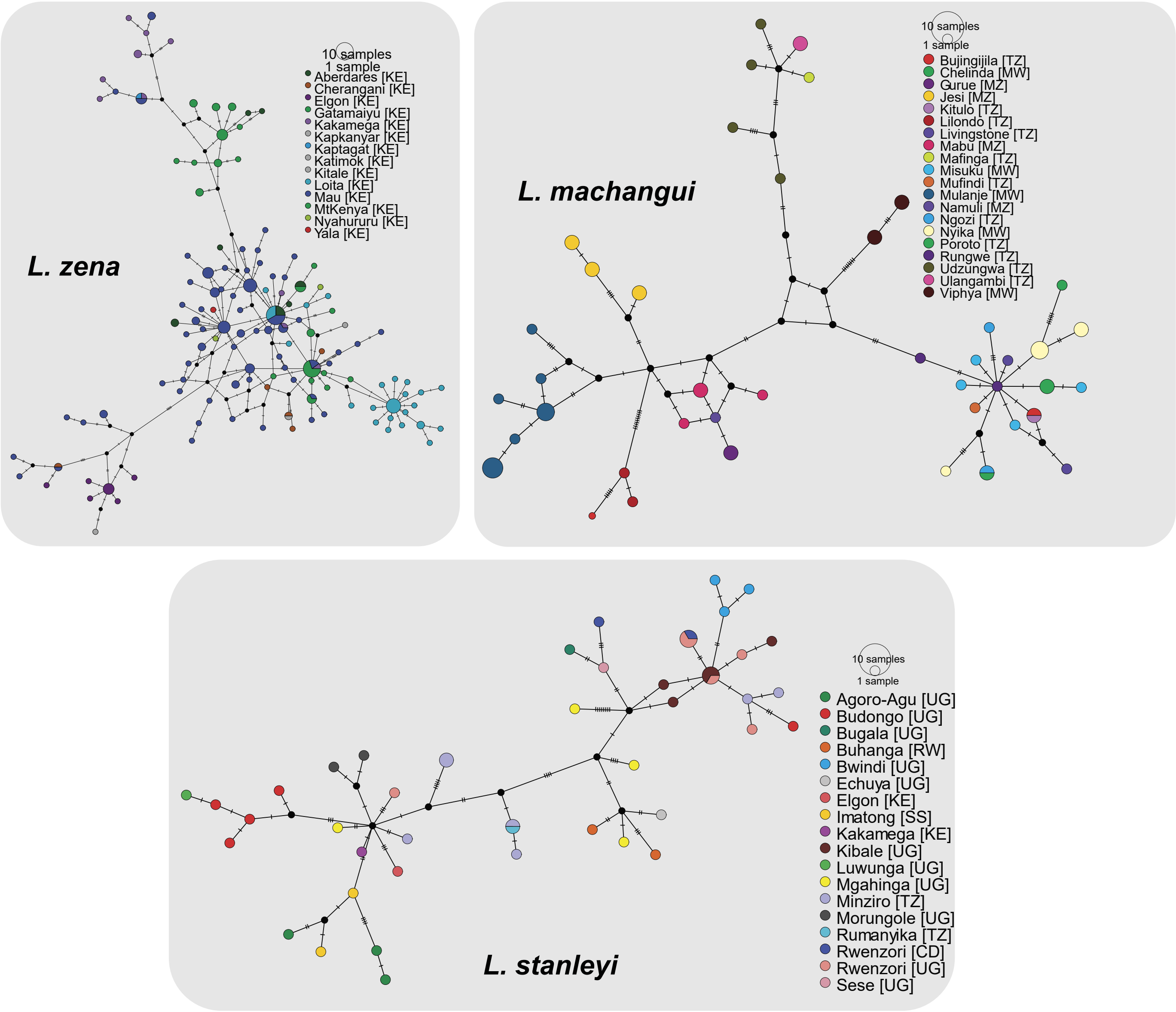

Fig. S6 Time calibrated maximum clade credibility tree of evolutionary relationships and divergence times of the non-Ethiopian *L. flavopunctatus* members reconstructed from concatenated alignments of *Cytochrome b* (*CYTB*)*, cytochrome oxidase subunit 1*, and *Interphotoreceptor retinoid binding protein*, time-callibrated using most recent common ancestor ages. Branch labels show the posterior probability support values and node bars show the highest posterior density interval. The corresponding gene trees are also presented to illustrate the phylogenetic informativeness of the individual genes. Here it is apparent that only the *CYTB* topology offer a reliable outlook of the phylogenetic associations between the non-Ethiopian *L. flavopunctatus* members.

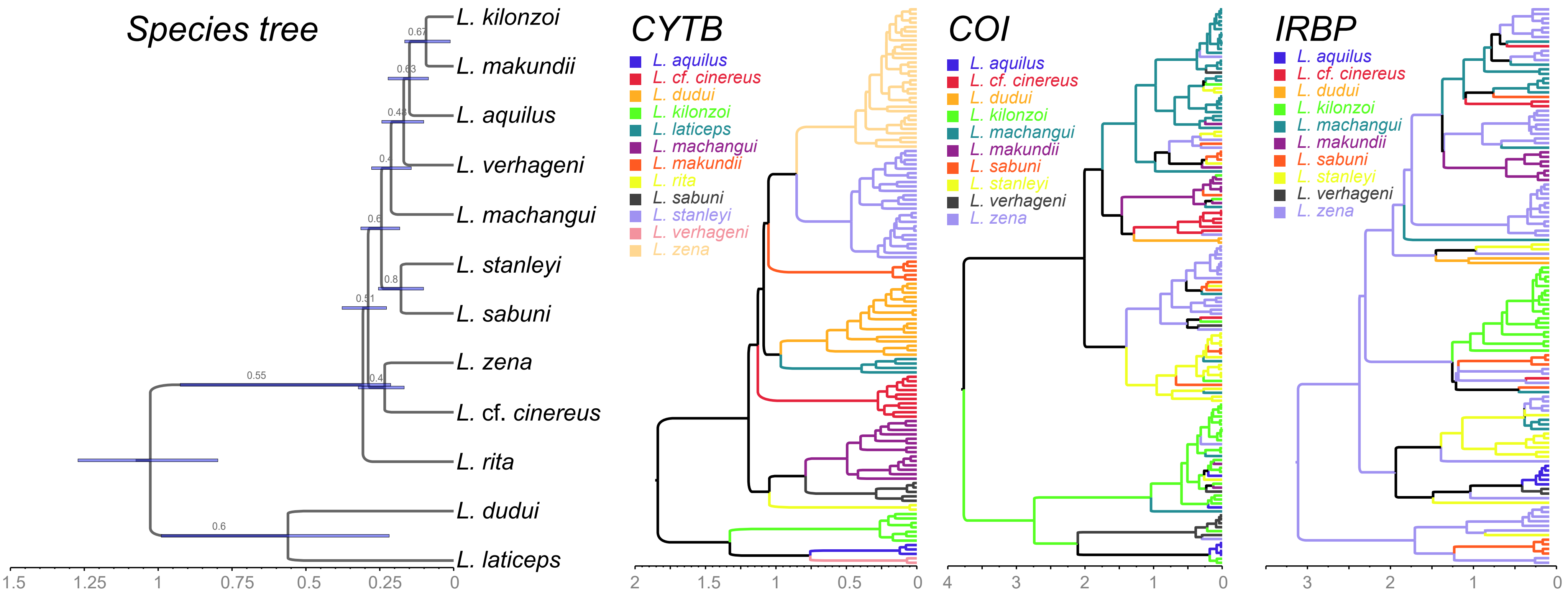

Fig. S7 Classification results following discriminant analysis in using linear and gometric craniodental datasets of the non-Ethiopian *L. flavopunctatus* members. Values indicate cross-validated (leave-one-our bootstrapping) percentage success by which samples were classified into a priori and predicted species groups. The shaded diagonal values indicate the success by which samples were predicted into their own groups which correspond to distinct *Cytochrome b* clades. N = number of samples. See **Fig. 6** in main manuscript for stratified species classification results.

Dataset	Species	1	2	3	4	5	6	7	8	9	10	11	N
Linear	1 *L. aquilus*	92.3	0.0	0.0	0.0	0.0	0.0	0.0	0.0	0.0	7.7	0.0	13
	2 *L.* cf. *cinererus*	4.9	47.5	3.3	8.2	18.0	4.9	4.9	0.0	6.6	1.6	0.0	61
	3 *L. dudui*	0.0	13.8	55.2	3.4	17.2	0.0	0.0	0.0	6.9	0.0	3.4	29
	4 *L. kilonzoi*	1.4	12.2	2.7	35.1	14.9	12.2	4.1	0.0	9.5	2.7	5.4	74
	5 *L. laticeps*	0.0	23.1	3.8	11.5	38.5	3.8	0.0	3.8	15.4	0.0	0.0	26
	6 *L. machangui*	1.2	4.7	4.7	11.8	1.2	52.9	0.0	9.4	4.7	1.2	8.2	85
	7 *L. makundii*	0.0	3.3	0.0	0.0	0.0	0.0	83.3	0.0	6.7	0.0	6.7	30
	8 *L. sabuni*	0.0	0.0	0.0	9.1	0.0	9.1	0.0	77.3	0.0	0.0	4.5	22
	9 *L. stanleyi*	1.4	11.8	11.4	10.0	11.4	5.7	7.1	1.4	29.9	0.5	9.5	211
	10 *L. verhageni*	0.0	0.0	0.0	0.0	0.0	0.0	5.9	0.0	0.0	94.1	0.0	17
	11 *L. zena*	1.3	2.5	10.8	5.7	6.4	12.1	19.1	5.7	7.6	2.5	26.1	157
Geometric	1 *L. aquilus*	100	0	0	0	0	0	0	0	0	0	0	13
	2 *L.* cf. *cinereus*	0	52.6	8.8	1.8	7	5.3	5.3	0	17.5	0	1.8	57
	3 *L. dudui*	0	20.7	55.2	0	6.9	0	0	3.4	13.8	0	0	29
	4 *L. kilonzoi*	0	1.5	1.5	74.6	0	1.5	1.5	0	10.4	1.5	7.5	67
	5 *L.* laticeps	0	14.3	9.5	4.8	42.9	0	0	4.8	14.3	0	9.5	21
	6 *L. machangui*	2.5	0	2.5	5.1	0	75.9	1.3	6.3	2.5	1.3	2.5	79
	7 *L. makundii*	0	0	3.6	3.6	0	0	82.1	0	0	0	10.7	28
	8 *L. sabuni*	0	0	0	21.1	0	21.1	0	47.4	0	0	10.5	19
	9 *L. stanleyi*	0	12.1	5.5	8.5	8	3.5	3	0.5	48.7	0.5	9.5	199
	10 *L. verhageni*	0	6.7	0	0	0	0	0	6.7	0	80	6.7	15
	11 *L. zena*	2.8	1.9	1.9	6.5	5.6	5.6	7.5	3.7	6.5	4.7	53.3	107

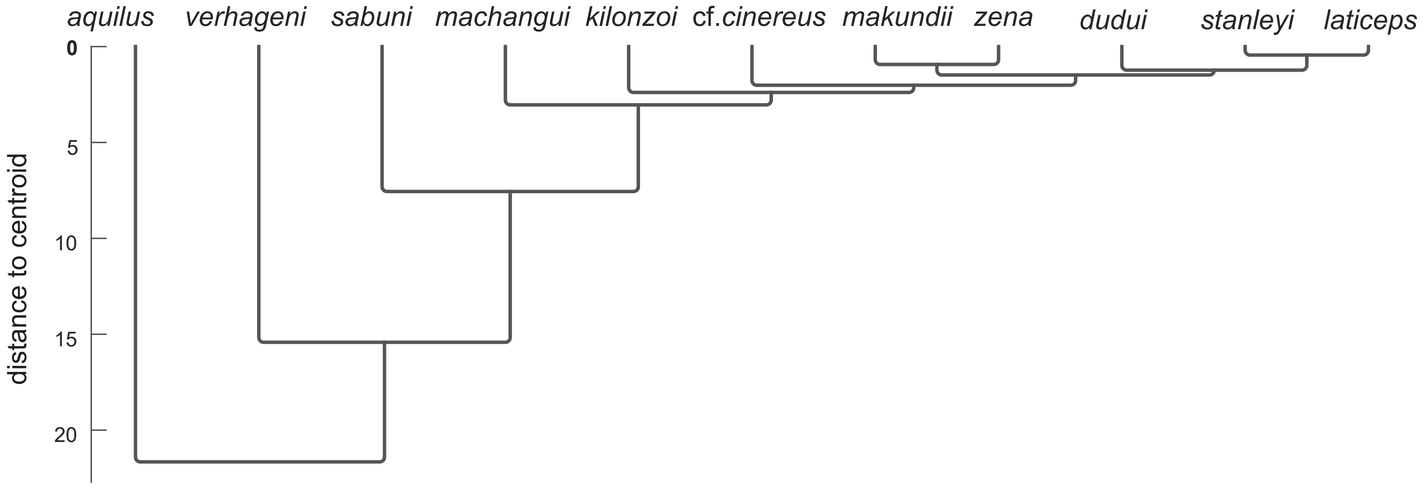

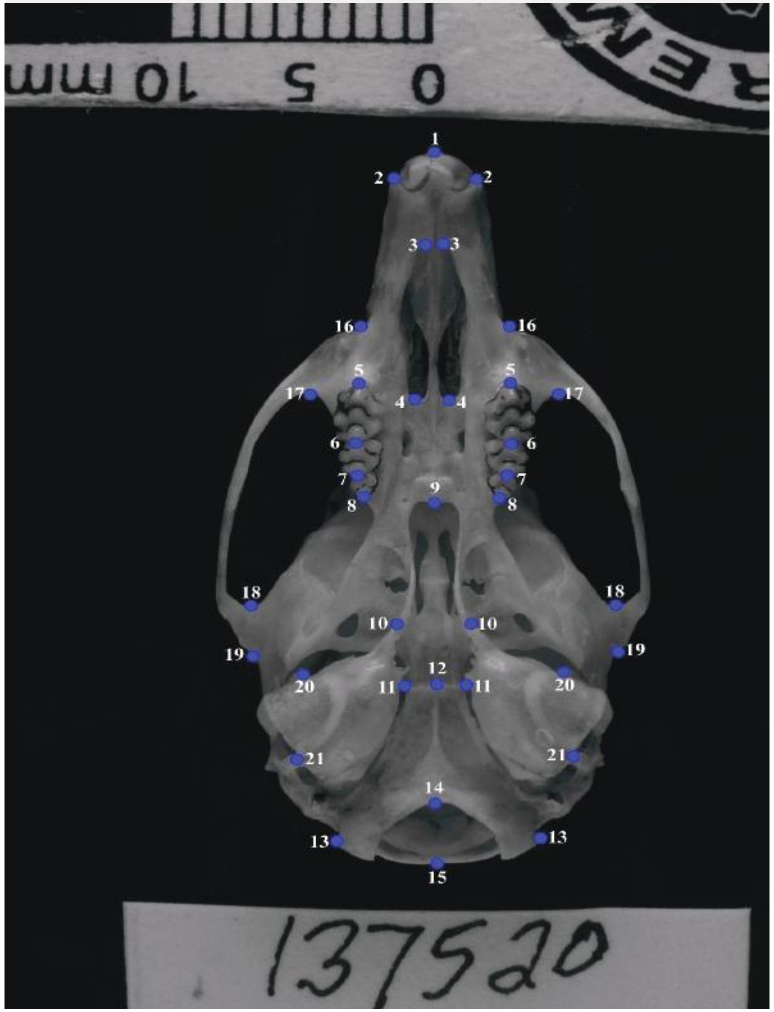

Fig. S8 Cranial landmarks used in geometric morphometric analyses of the non-Ethiopian *L. flavopunctatus* members. 1, Anteriormost point between upper incisors; 2, Superiormost point of upper incisive alveolus; 3, Anterior most margin of the incisive foramen; 4, Posteriormost margin of incisive foramen; ; 5, Anterior most margin of the first molar; 6, Between first molar and second molar; 7, Between the second molar and third molar; 8, Posteriormost margin of the third molar; 9, Posterior most point of the palate; 10, midpoint on the auditory bulla where it meets the piriform fenestra posteriorly; 11, Medial point of the junction between tympanic bullae and Eustachian tube; 12, Midpoint of suture between basisphenoid and basiocciptal; 13, Posteriormost margin of occipital condyle; 14, Anterior most point of the Foramen magnum viewed ventrally; 15, Posteriormost point of superior margin of foramen magnum; 16, Anteriormost margin of zygomatic plate; 17, Posteriormost margin of the posterior zygomatic plate; 18, Posteriormost margin of the anterior zygomatic plate; 19, Posterior most point of suture between jugal and squamosal; 20, Tip of the eustachian tube; 21, caudal end of the external opening of the auditory channel.

Fig. S9 Principal component analysis (PCA) of linear measurements used in the study. The plots show how samples cluster based on the first and second component scores. The top left scatter plot shows samples do not cluster in a distinct pattern consistent with the taxonomic units currently acknowledged in literature (top right). The bottom left plot shows the variances accounted for by all the component loadings.

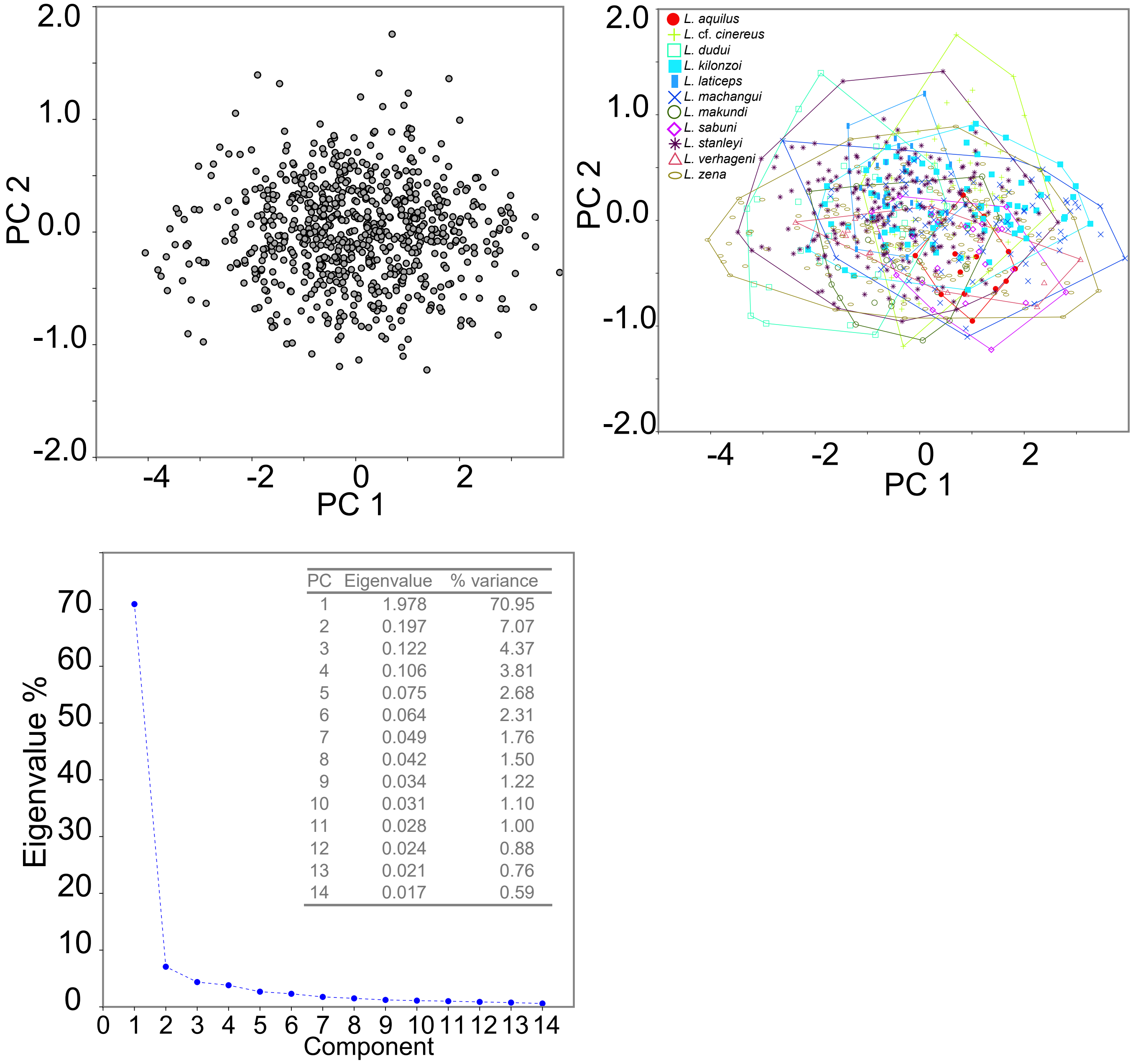

PC	Eigenvalue	% variance
1	1.978	70.95
2	0.197	7.07
3	0.122	4.37
4	0.106	3.81
5	0.075	2.68
6	0.064	2.31
7	0.049	1.76
8	0.042	1.50
9	0.034	1.22
10	0.031	1.10
11	0.028	1.00
12	0.024	0.88
13	0.021	0.76
14	0.017	0.59
