# Supplementary material for: Historical biogeography, systematics, and integrative taxonomy of the non-Ethiopian speckled pelage brush-furred rats (Lophuromys flavopunctatus group)
Source: BMC Ecol Evol. 2021 May 19;21:89. doi: 10.1186/s12862-021-01813-w (PMC8132446; doi:10.1186/s12862-021-01813-w)
Supplement: Supplementary file 2 — Additional file 2: Table S1. Summary statistics of linear cranial measurements and centroid size (CS) of species the non-Ethiopian L. flavopunctatus members. The values represent the 95% confidence interval mean | standard deviation | minimum–maximum values. Table S2. Summary of multivariate pairwise differences between the non-Ethiopian L. flavopunctatus members based on linear measurements and geometric craniodental landmarks inferred using permutational multivariate analysis of variance. Values represent pairwise PerMANOVAs between clade pairs as post hoc tests with the upper matrix showing the F values and the lower matrix showing the Bonferroni-corrected p values (statistically significant values are in bold). The test of PerMANOVA showed overall significant skull differences between clades in both the linear dataset (Total sum of squares: 35510; Within-group sum of squares: 29300; F: 15.13, p: 0.0001) and geometric dataset (Total sum of squares:0.3591; Within-group sum of squares: 0.3091; F: 10.1; p: 0.0001). Table S3. List of genes amplified showing the respective primers used and PCR reaction settings. F = forward primer, R = reverse primer. The thermal profile for IRBP amplification consisted of a touch-down annealing protocol from 52 to 56 °C. [file 12862_2021_1813_MOESM2_ESM.pdf]

Table S1 summary statistics of linear cranial measurements and centroid size (CS) of species ascribed to the non-Ethiopian L. flavopunctatus members. The values represent the mean ± standard deviation and minimum(Max)-maximum(Min) values. The *F* statistics and *p* values show the statistical significances of differences between the clades derived using analysis of variance in IBM SPSS Statistics v25. Refer to Onditi et. al., (2019)* for an illustration of the measurements.

		CI		ZB		BBC		PPL		LD		BOC		BM1	
Clade	N	Mean ± SD	Min. - Max.	Mean ± SD	Min. - Max.	Mean ± SD	Min. - Max.	Mean ± SD	Min. - Max.	Mean ± SD	Min. - Max.	Mean ± SD	Min. - Max.	Mean ± SD	Min. - Max.
L. aquilus	13	29.26 ± 0.45	28.41 - 29.85	14.87 ± 0.34	14.37 - 15.44	12.98 ± 0.24	12.64 - 13.42	12.48 ± 0.25	12.10 - 13.00	8.73 ± 0.18	8.40 - 9.09	7.19 ± 0.17	6.89 - 7.48	6.72 ± 0.09	6.58 - 6.88
L. verhageni	15	28.70 ± 1.23	26.59 - 31.01	14.50 ± 0.49	13.89 - 15.51	12.90 ± 0.30	12.32 - 13.52	12.12 ± 0.59	11.33 - 13.20	8.04 ± 0.54	7.16 - 9.05	7.30 ± 0.23	7.00 - 7.77	6.64 ± 0.58	5.09 - 7.22
L. kilonzoi	67	28.81 ± 0.97	26.94 - 30.78	15.10 ± 0.62	13.62 - 16.34	12.96 ± 0.31	12.30 - 13.62	12.22 ± 0.58	10.99 - 13.27	8.18 ± 0.39	7.47 - 9.32	7.30 ± 0.28	6.34 - 7.92	6.94 ± 0.23	6.52 - 7.45
L. cf. *cinereus*	57	28.72 ± 0.91	26.21 - 30.93	15.21 ± 0.58	13.71 - 16.38	13.14 ± 0.36	12.48 - 13.96	12.26 ± 0.52	10.78 - 13.70	8.19 ± 0.41	6.98 - 9.10	7.23 ± 0.23	6.72 - 7.65	6.89 ± 0.26	6.30 - 7.47
L. dudui	29	27.22 ± 0.88	25.82 - 28.70	14.29 ± 0.62	12.81 - 15.20	12.59 ± 0.48	11.85 - 13.34	11.59 ± 0.49	10.60 - 12.55	7.80 ± 0.34	7.16 - 8.52	7.01 ± 0.24	6.63 - 7.53	6.67 ± 0.27	6.15 - 7.21
L. laticeps	21	28.24 ± 0.93	27.04 - 30.98	14.93 ± 0.48	14.10 - 15.98	13.06 ± 0.26	12.50 - 13.79	11.90 ± 0.52	11.20 - 13.56	8.02 ± 0.33	7.43 - 8.79	7.25 ± 0.20	6.95 - 7.77	6.85 ± 0.31	6.25 - 7.71
L. machangui	79	29.07 ± 1.06	26.24 - 31.55	15.14 ± 0.56	13.92 - 16.34	12.92 ± 0.30	12.18 - 13.58	12.25 ± 0.54	10.71 - 13.76	8.39 ± 0.44	7.24 - 9.25	7.28 ± 0.26	6.79 - 7.93	7.09 ± 0.26	6.59 - 7.71
L. makundii	28	28.36 ± 0.72	26.85 - 29.55	14.74 ± 0.52	13.73 - 15.84	12.45 ± 0.35	11.85 - 13.11	11.97 ± 0.39	11.16 - 12.83	8.01 ± 0.31	7.46 - 8.61	6.99 ± 0.18	6.53 - 7.31	6.56 ± 0.21	6.17 - 6.95
L. sabuni	19	29.18 ± 0.71	27.75 - 30.81	14.83 ± 0.48	14.12 - 16.11	12.96 ± 0.25	12.43 - 13.38	12.20 ± 0.54	11.09 - 13.30	8.33 ± 0.29	7.90 - 9.08	7.35 ± 0.16	7.08 - 7.66	7.04 ± 0.23	6.63 - 7.50
L. stanleyi	199	27.99 ± 0.98	24.79 - 31.43	14.72 ± 0.57	13.12 - 16.67	12.81 ± 0.35	12.00 - 13.89	11.82 ± 0.56	10.11 - 13.60	7.98 ± 0.40	6.41 - 8.98	7.13 ± 0.24	6.60 - 8.09	6.77 ± 0.27	6.09 - 7.80
L. zena	108	28.32 ± 1.23	25.27 - 31.41	14.72 ± 0.70	12.73 - 16.62	12.69 ± 0.38	11.43 - 13.71	11.80 ± 0.71	10.00 - 14.02	8.12 ± 0.48	6.96 - 9.13	7.13 ± 0.27	6.49 - 7.80	6.76 ± 0.28	6.06 - 7.43
Statistics		$F = 15.197, p = 0.000$		$F = 10.576, p = 0.000$		$F = 15.949, p = 0.000$		$F = 10.258, p = 0.000$		$F = 11.824, p = 0.000$		$F = 9.882, p = 0.000$		$F = 17.069, p = 0.000$	

		LIF		IOB		LAB		ALM		BR		LBP		BZP		CS	
Clade	N	Mean ± SD	Min. - Max.	Mean ± SD	Min. - Max.	Mean ± SD	Min. - Max.	Mean ± SD	Min. - Max.	Mean ± SD	Min. - Max.	Mean ± SD	Min. - Max.	Mean ± SD	Min. - Max.	Mean ± SD	Min. - Max.
L. aquilus	13	6.74 ± 0.28	6.30 - 7.24	6.05 ± 0.12	5.80 - 6.26	5.48 ± 0.12	5.27 - 5.66	4.72 ± 0.14	4.45 - 4.94	5.06 ± 0.18	4.77 - 5.38	4.15 ± 0.21	3.76 - 4.39	2.80 ± 0.10	2.64 - 2.97	56.88 ± 0.87	55.12 - 57.93
L. verhageni	15	6.01 ± 0.29	5.51 - 6.51	6.09 ± 0.18	5.80 - 6.41	5.48 ± 0.24	5.18 - 5.92	5.03 ± 0.10	4.79 - 5.25	4.86 ± 0.17	4.58 - 5.25	4.73 ± 0.29	4.31 - 5.22	2.83 ± 0.13	2.65 - 3.13	56.08 ± 2.60	52.16 - 60.95
L. kilonzoi	67	6.42 ± 0.31	5.63 - 7.01	6.34 ± 0.23	5.81 - 6.98	5.36 ± 0.20	4.94 - 5.78	4.96 ± 0.20	4.39 - 5.35	5.01 ± 0.21	4.58 - 5.52	4.17 ± 0.28	3.58 - 5.05	3.10 ± 0.19	2.73 - 3.53	56.67 ± 2.00	52.45 - 60.25
L. cf. *cinereus*	57	6.45 ± 0.35	5.80 - 7.42	6.31 ± 0.25	5.63 - 7.09	5.47 ± 0.21	5.05 - 5.86	4.87 ± 0.21	4.53 - 5.40	5.01 ± 0.23	4.56 - 5.53	4.02 ± 0.28	3.41 - 4.54	2.91 ± 0.19	2.46 - 3.46	56.16 ± 1.78	51.04 - 60.60
L. dudui	29	6.27 ± 0.32	5.58 - 7.04	5.88 ± 0.26	5.36 - 6.31	5.20 ± 0.25	4.85 - 5.78	4.72 ± 0.22	4.27 - 5.17	4.73 ± 0.19	4.36 - 5.09	3.76 ± 0.20	3.32 - 4.08	2.81 ± 0.12	2.52 - 3.01	53.28 ± 1.71	50.63 - 56.26
L. laticeps	21	6.37 ± 0.25	5.87 - 6.91	6.23 ± 0.19	5.82 - 6.54	5.38 ± 0.20	4.96 - 5.73	5.00 ± 0.19	4.73 - 5.44	4.87 ± 0.19	4.47 - 5.36	4.04 ± 0.26	3.51 - 4.64	2.95 ± 0.19	2.64 - 3.45	55.08 ± 1.17	53.72 - 58.99
L. machangui	79	6.64 ± 0.34	5.78 - 7.37	6.21 ± 0.22	5.71 - 6.95	5.28 ± 0.17	4.90 - 5.74	5.09 ± 0.17	4.58 - 5.53	5.05 ± 0.24	4.42 - 5.79	4.19 ± 0.33	3.37 - 5.09	3.12 ± 0.18	2.68 - 3.58	56.82 ± 1.94	52.05 - 60.86
L. makundii	28	6.20 ± 0.26	5.49 - 6.67	5.96 ± 0.17	5.68 - 6.36	5.47 ± 0.19	5.10 - 5.83	4.87 ± 0.16	4.49 - 5.16	4.81 ± 0.20	4.45 - 5.23	4.27 ± 0.27	3.84 - 4.89	3.08 ± 0.14	2.72 - 3.36	55.47 ± 1.50	52.58 - 57.94
L. sabuni	19	6.73 ± 0.25	6.31 - 7.31	6.09 ± 0.16	5.78 - 6.48	5.35 ± 0.18	5.02 - 5.78	5.20 ± 0.21	4.79 - 5.57	4.88 ± 0.19	4.48 - 5.26	4.38 ± 0.26	3.85 - 4.79	3.21 ± 0.15	3.00 - 3.65	57.15 ± 1.30	54.68 - 59.89
L. stanleyi	199	6.30 ± 0.36	5.39 - 7.58	6.15 ± 0.25	5.48 - 6.92	5.36 ± 0.20	4.90 - 5.93	4.81 ± 0.24	4.09 - 5.77	4.84 ± 0.26	4.26 - 5.74	4.03 ± 0.28	3.33 - 4.77	3.04 ± 0.22	2.59 - 3.67	54.98 ± 1.88	49.54 - 61.47
L. zena	108	6.43 ± 0.36	5.57 - 7.22	6.02 ± 0.26	5.36 - 6.81	5.41 ± 0.24	4.87 - 6.00	4.94 ± 0.23	4.45 - 5.52	4.85 ± 0.27	4.26 - 5.50	4.20 ± 0.30	3.48 - 5.09	3.07 ± 0.25	2.43 - 3.77	55.60 ± 2.60	49.35 - 60.72
Statistics		$F = 13.513, p = 0.000$		$F = 19.313, p = 0.000$		$F = 6.893, p = 0.000$		$F = 20.197, p = 0.000$		$F = 10.530, p = 0.000$		$F = 19.957, p = 0.000$		$F = 14.276, p = 0.000$		$F = 13.078, p = 0.000$	

CS, centroid size; CI, Condylo-insicive length; ZB, Greatest zygomatic breadth; BBC, Breadth of braincase; BOC, Breadth across the occipital condyles; LOB, Least interorbital breadth; BR, Greatest breadth of rostrum; PPL, Postpalatal length; LBP, Length of bony palate; LIF, Length of incisive foramen; LD, Length of diastema; BZP, Breadth of zygomatic plate; LAB, Length of auditory bulla; ALM, Alveolar length of the maxillary toothrow; BM1, coronal breadth of the first upper molar. N = number of samples.

* Onditi KO, Kerbis Peterhans JC, Demos TC, Musila S, Zhongzheng C, Xuelong J. Morphological and genetic characterization of Mount Kenya brush-furred rats (*Lophuromys* Peters 1874); relevance to taxonomy and ecology. Mammal Research 2019, 65(2):387-400; https://doi.org/10.1007/s13364-019-00470-1.

Table S2 Summary of multivariate pairwise differences between the non-Ethiopian *L. flavopunctatus* members based on linear measurements and geometric craniodental landmarks inferred using permutational multivariate analysis of variance (PerMANOVA). Values represent pairwise PerMANOVAs between clade pairs as post hoc tests with the upper matrix showing the F values and the lower matrix showing the Bonferroni-corrected p values (statistically significant values are in bold). The test of PerMANOVA showed overall significant skull differences between clades in both the linear dataset (Total sum of squares: 35510; Within-group sum of squares: 29300; F : 15.13, p : 0.0001) and geometric dataset (Total sum of squares:0.3591; Within-group sum of squares: 0.3091; F: 10.1; p: 0.0001).

Linear dataset

	**1**	**2**	**3**	**4**	**5**	**6**	**7**	**8**	**9**	**10**	**11**
**1** *L. aquilus*		6.578	23.54	8.012	22.73	7.719	18.17	12.37	12.78	9.963	7.265
**2** *L.* cf. *cinereus*	**0.011**		39.89	3.861	9.075	11.97	24	10.86	24.99	8.876	18.88
**3** *L. dudui*	**0.0055**	**0.0055**		39.81	13.92	52.41	10.67	32.09	13.26	12.05	12.3
**4** *L. kilonzoi*	**0.011**	0.6655	**0.0055**		8.675	5.845	21.13	5.242	25.38	7.888	17.46
**5** *L. laticeps*	**0.0055**	**0.0055**	**0.011**	**0.0165**		17.75	12.43	17.36	2.296	6.108	3.527
**6** *L. machangui*	**0.0385**	**0.0055**	**0.0055**	0.22	**0.0055**		30.86	2.927	50.26	12.75	28.73
**7** *L. makundii*	**0.0055**	**0.0055**	**0.0055**	**0.0055**	**0.0055**	**0.0055**		21.94	6.208	7.403	2.982
**8** *L. sabuni*	**0.0055**	**0.0055**	**0.0055**	0.2255	**0.0055**	1	**0.0055**		16.53	8.911	8.435
**9** *L. stanleyi*	**0.0055**	**0.0055**	**0.0055**	**0.0055**	1	**0.0055**	0.077	**0.0055**		6.275	5.924
**10** *L. verhageni*	**0.011**	**0.0055**	**0.0055**	**0.0385**	**0.033**	**0.0055**	**0.022**	**0.011**	0.0715		4.042
**11** *L. zena*	0.088	**0.0055**	**0.0055**	**0.0055**	1	**0.0055**	1	**0.0275**	0.0825	1	

Geometric dataset

	**1**	**2**	**3**	**4**	**5**	**6**	**7**	**8**	**9**	**10**	**11**
**1** *L. aquilus*		10.18	6.423	13.64	9.309	8.911	14.16	10.31	12.31	9.511	11.68
**2** *L.* cf. *cinereus*	**0.0055**		4.836	13.09	1.974	15.92	14.2	10.38	7.078	10.09	12.57
**3** *L. dudui*	**0.0055**	**0.0055**		9.227	3.336	6.477	13.22	6.534	5.374	10.87	8.529
**4** *L. kilonzoi*	**0.0055**	**0.0055**	**0.0055**		5.197	10.96	17.47	6.518	9.718	12.32	17.82
**5** *L. laticeps*	**0.0055**	0.6105	**0.011**	**0.0055**		5.546	9.725	6.097	2.018	8.921	3.43
**6** *L. machangui*	**0.0055**	**0.0055**	**0.0055**	**0.0055**	**0.0055**		21.04	4.458	18.78	11.66	16.91
**7** *L. makundii*	**0.0055**	**0.0055**	**0.0055**	**0.0055**	**0.0055**	**0.0055**		9.826	11.6	7.296	7.805
**8** *L. sabuni*	**0.0055**	**0.0055**	**0.0055**	**0.0055**	**0.0055**	**0.0055**	**0.0055**		7.394	8.679	5.877
**9** *L. stanleyi*	**0.0055**	**0.0055**	**0.0055**	**0.0055**	0.627	**0.0055**	**0.0055**	**0.0055**		11.8	9.84
**10** *L. verhageni*	**0.0055**	**0.0055**	**0.0055**	**0.0055**	**0.0055**	**0.0055**	**0.0055**	**0.0055**	**0.0055**		10.32
**11** *L. zena*	**0.0055**	**0.0055**	**0.0055**	**0.0055**	**0.0055**	**0.0055**	**0.0055**	**0.0055**	**0.0055**	**0.006**	

Table S3 List of genes amplified showing the respective primers used and PCR reaction settings. F = forward primer, R = reverse primer. The thermal profile for *IRBP* amplification consisted of a touch-down annealing protocol from 52 to 56 °C. Primer references: *CYTB* (Kocher *et al.*, 1989), *COI* (Robins *et al.*, 2007), and *IRBP* (Stanhope *et al.*, 1996).

Loci		Primer Sequence	*De*	*An*	*Ex*	*Cy*
CYTB	*F*	GGACTTATGACATGAAAAATCATCGTTG	30s, 95°C	30s, 59°C	60s, 72°C	40
	R	GATTCCCCATTTCTGGTTTACAAGAC				
COI	*F*	CCTACTCRGCCATTTTACCTATG	30s, 95°C	30s, 56°C	30s, 72°C	35
	R	ACTTCTGGGTGTCCAAAGAATCA				
IRBP	*F*	ATGGCCAAGGTCCTCTTGGATAACTACTGCTT	20s, 94°C	20s, 52°C	80s, 72°C	10
	R	CGCAGGTCCATGATGAGGTBCTCCGTGTCCTG	20s, 94°C	20s, 56°C	80s, 72°C	20

References

Kocher, T. D., et al. 1989. Dynamics of mitochondrial DNA evolution in animals: amplification and sequencing with conserved primers. Proc Natl Acad Sci U S A 86:6196-6200.

Robins, J. H., Hingston, M., MATISOO-SMITH, E., & Ross, H. A. 2007. Identifying Rattus species using mitochondrial DNA. Molecular ecology notes 7:717-729.

Stanhope, M. J., Smith, M. R., Waddell, V. G., Porter, C. A., Shivji, M. S., & Goodman, M. 1996. Mammalian evolution and the interphotoreceptor retinoid binding protein (IRBP) gene: convincing evidence for several superordinal clades. J Mol Evol 43:83-92.
